# Mononostril versus Binostril Endoscopic Transsphenoidal Approach for Pituitary Adenomas: A Systematic Review and Meta-Analysis

**DOI:** 10.1371/journal.pone.0153397

**Published:** 2016-04-28

**Authors:** Guodao Wen, Chao Tang, Chunyu Zhong, Xiang Li, Junyang Li, Liwen Li, Youqing Yang, Chiyuan Ma

**Affiliations:** 1 Department of Neurosurgery, Jinling Hospital, School of Medicine, Nanjing University, 305 East Zhongshan Road, Nanjing, 210002, China; 2 School of Medicine, Nanjing Medical University, 104 Hanzhong Road, Nanjing, 210002, China; 3 School of Medicine, Southern Medical University, 1813 North Guangzhou Big Road, Guangzhou, 510000, China; Universita' degli Studi di Napoli Federico II, ITALY

## Abstract

**Background:**

Over the past several decades, the endoscopic endonasal transsphenoidal approach (EETA) has gradually become a preferred option of pituitary adenomas surgery because of its minimal invasiveness and high efficiency. However, some EETA operations were performed through one nostril (mononostril), while other EETA operations were performed through both nostrils (binostril). Therefore, we conducted this study to compare the pros and cons of these two methods in an attempted to confirm which method is more effective.

**Methods:**

We executed a systematic literature search of PubMed, the Cochrane Library, and the Web of Science and Medline (1992–2015). The language is limited to English and all studies should meet the inclusion criteria. Comparisons were made for postoperative outcomes, complications, and other relevant parameters between the mononostril and the binostril group. Statistical analyses of categorical variables were undertaken by the use of Stata 12.0 and SPASS 19.0.

**Results:**

Thirty studies, involving 4805 patients, were included. The two groups had similar results in GTR rate (included GTR rate of macroadenomas), hormonal remission rate, improvement in visual function, postoperative CSF leak, permanent diabetes insipidus, meningitis, and sinusitis. The binostril group had less temporary diabetes insipidus (2.9% vs. 5.3%, p = 0.022), less anterior pituitary insufficiency (2.3% vs. 6.4%, p = 0.000) and few hospitalization days (3.2 days vs. 4.4 days, p<0.05) than the mononostril group. However, the mononostril group had less rate of epistaxis (0.4% vs. 1.5%, p = 0.008) than the binostril group. For invasive macroadenomas, the binostril group seem to demonstrate a tendency towards better outcomes though there was no subgroup analysis between the two groups.

**Conclusion:**

The binostril approach had less temporary diabetes insipidus, anterior pituitary insufficiency, and a shorter length of hospital stay, although they demonstrated a higher rate of epistaxis than the mononstril group. Additionally, the binostril group seemed to suggest a tendency towards better outcomes for invasive macroadenomas.

## Introduction

The last decades have seen a rapid development and an ongoing refinement of the endoscopic transsphenoidal pituitary surgery, since Jankowski and co-workers first described a fully endoscopic approach to pituitary surgery in 1992 [1].Currently, the endoscopic transsphenoidal surgery has become the first option to resect pituitary adenomas, due to the wider visualization while being less invasive than ever before, [2–4], resulting in the safer extraction of pituitary adenomas. Traditionally, there are two routes of endoscopic endonasal transsphenoidal in regards to the pituitary adenomas, namely, one nostril (mononostril) and two nostrils (binostril) [5, 6]. Some clinical centers have advocated the binostril approach to increase the space of instrument manipulation, whereas other centers prefer the minimally invasive mononostril method. It remains unclear which is the best approach.

Therefore, we performed this meta-analysis to compare the postoperative outcomes, complications, and other relevant parameters between the mononostril and the binostril method in order to obtain a comprehensive evaluation of the benefits and limitations of these two approaches and facilitate the process of surgical method decision making.

## Methods

### Search strategy

A systematic search of PubMed, the Cochrane Library, the Web of Science, and Medline was performed for relevant literature dating from February 1992 to 2015 August. We recognized all relevant published and unpublished literature via a thorough search strategy. The key phrases were described in supporting material (S1 File).The search results were only included literature in English and humans for the study category. We reviewed all titles and abstracts, and each potentially relevant article was marked for further examine. The full-text articles of the marked studies were included if satisfied our inclusion criteria. Additionally, the references listed in all obtained studies were also screened for possible inclusion.

GDW did the systematic search, reviewed of all abstracts, marked relevant full-text articles. CT and CYZ independently evaluated the full texts according to the predefined inclusion criteria and screened the references listed in all obtained studies. If a full text was assessed by both authors and satisfied the inclusion criteria, they were included. Any disagreement was determined by a third reviewer CYM.

### Inclusion criteria

Studies were considered appropriate for inclusion if they met the following criteria: 1) adoption of either a purely mononostril or binostril endoscopic transsphenoidal approach and 2) series should include 50 or more patients with pituitary adenoma who had undergone surgery in the same center. A cutoff of 50 patients per approach was arbitrarily chosen to exclude those literatures presenting merely their preliminary experience with an approach. Series including variations of the endoscopic-assisted approach or microscopic approaches were excluded.

The items analyzed must include: 1) gross tumor resection (GTR): absence of residual tumor on postoperative MRI; 2) postoperative hormonal remission rate: for patients with somatotrophic tumor, the level of IGF-I norm suitable to sex and age, the level of GH in OGTT <1 ng/ml(2000 year criterion[7]) or < 0.4 ng/mL (2010 year criterion[8]); the PRL levels were normalization in PRL-secreting adenomas; for patients with Cushing disease, post-operative normalized serum ACTH, cortisol levels, and free cortisol level in 24-hour urine collection; 3) improvement in visual function base on comparing preoperative with postoperative visual acuity and visual field examination; 4) the length of hospital stay, and 5) complications: postoperative cerebrospinal fluid [CSF] leaks requiring treated with lumbar drain or second surgery, permanent and temporary diabetes insipidus [DI], anterior pituitary insufficiency, meningitis, epistaxis, or sinusitis. The following data items were also recorded but cannot be used for statistical analysis, due to the number of studies or incidence relevant these data was rare: mortality, the length of operation, visual recovered, hyposmia, cranial nerve injury, intraoperative blood loss, intracavernous carotid artery injury, and recurrence rate.

### Statistical analysis

This meta-analysis was done in Stata (version12.0; Stata Corporation, College Station, TX). Considering the articles included in our study were not possible to compare using the traditional meta-analysis method with relative risk or OR., the different parameters of the included studies were analyzed respectively. A random effects-model was conducted for meta-analyses, due to the obvious clinical heterogeneity and the heterogeneity ofvariance was estimated with the DerSimonian and Laird approach [9]. For the dichotomous outcomes, estimated proportions and 95% confidence interval were described. All proportions were compared between the mononostril and the binostril approach using binomial tests and were considered significant difference if the P value was <0.05. Timothy R. DeKlotz [10] and Mario Ammirati [11] used this method when comparing outcomes of endoscopic to microscopic pituitary adenoma surgery. In addition, the Mann-Whitney U test was used for the length of operation and T-test was used for patient characteristics (SPASS 19.0), while the P-value of <0.05 was considered statistically significant.

## Results

### Search results

From the searches of databases, 2075 studies were initially identified. Part of these studies were excluded as duplicates using reference manager EndNote, and the majority was excluded on the basis of the titles or abstracts, resulting in 107 potentially relevant articles. Subsequently, the full texts of these studies were obtained and reviewed thoroughly, where a further 77 articles did not meet the inclusion criteria. We also attempted to find additional eligible articles from the references from the included articles, but without success. Finally, 30 articles were selected in this meta-analysis. All articles were retrospective clinical researches, among them 13 articles reported outcomes of mononostril approaches, 15 articles described outcomes for binostril approaches, and two articles were directly comparing the mononostrill and the binostril approach. In total, the 30 articles included 4865 patients. A detailed flowchart and the characteristics of the included studies are summarized in S1 Fig and S1 Table, respectively.

### Patient characteristics

In the mononostril cohort, there were 2285 patients, mean 45.5 years of age, mean follow-up 30.9 months, 39% male, and proportion of macroadenomas tumors was 75%. In the binostril cohort, there were 2580 patients, mean 48.7 years of age, mean follow-up 35.1 months, 44% male, and proportion of macroadenomas tumors was 83%. We failed to discover any statistically significant difference in the two approaches regarding patient characteristics (S2 Table).

### Outcomes analyses

#### Tumor resection rate

Outcome of surgery was evaluated using meta-analysis techniques. Reviewing the baseline characteristics of the surgical approaches, twelve mononostril (n = 1902) and ten binostril (n = 1382) studies reported data on GTR. The random effects pooled estimates of GTR was achieved in 77.8% (95% confidence interval [CI], 72.1–83.4) and 80.3% (95% CI, 72.2–88.4) of patients in mononostril and binostril approach, respectively. The analysis revealed insignificant differences between the mononostril group and the binostril group (p = 0.07940, S2 and S3 Figs).

We also estimated GTR of pituitary macroadenomas (maximum diameter≥10mm). Five mononostril (n = 442) and four binostril (n = 747) studies reported data on GTR of macroadenomas. The random pooled estimates of proportions were 70.4% (95% CI, 52.6–88.2) in the mononostril group and 72.7% (95% CI, 56.6–88.7) in the binostril group. There was no statistically significant difference in the two groups as well (p = 0.253329, S4 and S5 Figs).

#### Hormonal remission rate

Ten mononostril (n = 1137) and thirteen binostril (n = 1116) studies reported data on postoperative hormonal remission rate. The random pool estimated the proportions to be 72.9% (95% CI, 64.4–81.4) in the mononostril group and 76% (95% CI, 69.9–82.1) in the binostril group. There was no statistically significant difference in the two groups (p = 0.121, S6 and S7 Figs). We also evaluated the postoperative hormonal remission rate of GH, PRL or ACTH secreting adenomas, respectively. For the hormonal remission rate of GH adenomas, seven mononostril (n = 415) and twelve binostril (n = 618) studies reported relevant data, with the random pool estimates being 73.4% (95% CI, 63.9–82.8) and 75.5% (95% CI, 68.3–82.7) in the mononostril and the binostril approach, respectively (S8 and S9 Figs). For hormonal remission rate of PRL adenomas, six mononostril (n = 501) and nine binostril (n = 175) studies reported relevant data, the random pooled estimates of proportions were achieved in 70.9% (95% CI, 56.3–85.5) and 73.1% (95% CI, 59.7–86.5) in mononostril and binostril approach, respectively (S10 and S11 Figs). For the hormonal remission rate of ACTH adenomas, six mononostril (n = 81) and ten binostril (n = 257) studies reported relevant data, the random pool estimates being 80.3% (95% CI, 66.6–94.0) and 80.9% (95% CI, 72.0–89.8) in the mononostril and the binostril approache, respectively (S12 and S13 Figs). We failed to discover any statistically significant difference in the two approaches regarding these postoperative hormonal remission rate (p>0.05).

#### Improvement in visual function

Seven mononostril (n = 409) and eight binostril (n = 660) studies reported data on improvement in visual function. The random pooled estimates of proportions were 91.1% (95% CI, 86.5–95.8) in the mononostril group and 88% (95% CI, 81.8–92.2) in the binostril group, There was no statistically significant difference in the two groups (p = 0.316, S14 and S15 Figs).

#### Neurological complications analyses

Twelve mononostril (n = 1949) and fifteen binostril (n = 2303) studies reported data on CSF leakage. The random pool estimates were shown in 2.9% (95% CI, 1.9–4.0) and 3.1% (95% CI, 2.1–4.0) in the mononostril and the binostril approach, respectively. There was no statistically significant difference in the two groups (p = 0.725, S16 and S17 Figs).

Nine mononostril (n = 856) and eleven binostril (n = 2027) studies reported data on temporary diabetes insipidus. The random pooled estimates of proportions were 5.3% (95% CI, 3.7–6.8) in mononostril group and 2.9% (95% CI, 1.5–4.2) in binostril group. The binostril group had less occurance of temporary diabetes insipidus than the mononostril approach (p = 0.0218, S18 and S19 Figs).

Nine mononostril (n = 856) and eleven binostril (n = 1814) studies reported data on permanent diabetes insipidus. The random pool estimates were shown in 1.0% (95% CI, 0.3–1.7) and 0.9% (95% CI, 0.3–1.5) in the mononostril and the binostril approach, respectively. There was no statistically significant difference in the two groups (p = 0.779, S20 and S21 Figs).

Ten mononostril (n = 1040) and nine binostril (n = 1816) studies reported data on anterior pituitary insufficiency. The random pool estimates were shown to be 6.4% (95% CI, 3.4–9.4) in the mononostril group and 2.3% (95% CI, 1.1–3.5) in the binostril group. The binostril group had less rate of anterior pituitary insufficiency than the mononostril approach (p = 0.000, S22 and S23 Figs).

Nine mononostril (n = 957) and seven binostril (n = 1538) studies reported data on meningitis. The random pool estimates were shown in 0.8% (95% CI, 0.3–1.4) and 0.8% (95% CI, 0.3–1.2) in the mononostril and the binostril approach, respectively. There was no statistically significant difference in the two groups (p> 0.05, S24 and S25 Figs).

#### Nasal complications analyses

Eight mononostril (n = 1603) and six binostril (n = 1236) studies reported data on sinusitis. The random pool estimates were shown were 0.3% (95% CI, 0.0–0.5) in the mononostril group and 0.9% (95% CI, 0.0–1.7) in the binostril group. There was no statistically significant difference in the two groups (p = 0.1024, S26 and S27 Figs).

Ten mononostril (n = 1712) and ten binostril (n = 1644) studies reported data on epistaxis. The random pool estimates were shown in 0.4% (95% CI, 0.1–0.7) and 1.5% (95% CI, 0.6–2.4) in the mononostril and the binostril approach, respectively. A higher rate of epistaxis was performed in the binostril group than the mononostril group (p = 0.008, S28 and S29 Figs).

#### Other parameters analyses

Four mononostril (n = 584) and four binostril (n = 439) studies reported data on the length of hospital stay. The mean time was 4.4 days (range, 1.6–6.5 days) and 3.2 days (range, 2.3–4.7 days) in the mononostril and the binostril approach, respectively. There was a statistically significant difference between the two groups (Mann-Whitney U test, p = 0.000, S3 Table).

## Discussion

### Mononostril or binostril

The primary goal of pituitary surgery is to resect the adenoma as much as possible while minimizing the extent of possible surgical trauma to patients. Compared to traditional microsurgery, endoscopic endonasal pituitary surgery provides a wider angle view, more flexible mobile and close-up shots of the anatomical structures with the ability to obtain significantly higher cure rate, have less complications, as well as shorter operation durations, less blood loss, and shorter hospital stays [12,13]. However, neurosurgeons are dissatisfied with the current situation, resulting in a desire to find a maximally effective surgery approach to resect pituitary adenomas. Some doctors advocate the mononostril approach without crossing the nasal septum to limit the trauma of nose, whereas other doctors prefer the binostril approach to fully exposes the anatomy of the sella turcica and magnify the operative field. The demerits and merits regarding these two wide used surgery approaches have become a hot topic of discussion.

### For general pituitary adenomas

This meta-analysis did not demonstrate any significant difference between the mononostril and the binostril approaches within GTR (included GTR of macroadenomas), hormonal remission rate, improvement in visual function, CSF leak, temporary diabetes insipidus, and meningitis. Recognizing the limitations of a meta-analysis in drawing firm conclusions, we assumed it could be the following reasons: 1) advanced endoscopic equipment: as the development of neuroendoscopic technique continues, the high resolution endoscope can provide an excellent panoramic view of the surgical field and enhance the chance of total resection of the tumor [14–16]. Most surgeries were performed with the use of different angled endoscopes for visualization of the parasellar and suprasellar areas, thus allowing surgeons to identify clearly anatomic structures even in a very limited operative field [2,3, 17–25]; 2) Improvements of mononostril approach: although in the mononostril approach, it was unnecessary did no need to cross the nasal septum, many surgeons usuallydetached and pushed the nasal septum to the contralateral nasal cavity to obtain adequate operating space[6,15,17,22,23,25]. Additionally, some surgeons used a speculum to narrow the surgical corridor [2]; 3) other reasons: such as the cooperation with otorhinolaryngologist, the use of intraoperative navigation system or MRI [3,15,23], the characteristic of pituitary adenomas (the majority is soft). All these factors could increase the efficiency of the mononostril approach surgery. These aforementioned parameters demonstrate that the mononostril approach is able to cope with most pituitary adenomas. In other words, the advantage of the binostril approach is unclear within general pituitary adenomas.

In spite of this, this meta-analysis found that the binostril approach had less anterior pituitary insufficiency and temporary diabetes insipidus than mononostril approach. In our opinion, crossing the middle turbinate could enlarge the operational space and improve the view of surgical field to a certain degree; this advantage could result in more freedom of movement with the instruments and makes surgeons more comfortable. Therefore, the binostril approach could make increase identification clarity within the structure while minimizing trauma of the normal pituitary gland.

### For invasive pituitary adenomas

Han et al. reported that the rate of GTR was 0% in mononostril group and 28.6% in binostril group (P>0.05) for macroadenomas with Hardy grade 4 and/or Knosp grade 3 and 4 [6]. Linsler et al reported that GTR was 0% in mononostril approach for patients with invasive and giant tumors [4]. Dallapiazza et al reported that rate of GTR was 28% in binostril approach for patients with tumors with Knosp grade 3 and 4 [26]. Oertel et al [2] used the binostril approach for better visualization and to improve the manipulation of the lesion only in few cases: one clivus chordoma, one prepontine arachnoidal cyst and one brainstem cavernous malformation. Rudnik, A et al [20] and D'Haens, Jean et al [21] reported that GH hormonal remission rates were 8.3% (1/12) and 40% (2/5) in the mononostril approach for tumors with Knosp grade 3 and 4, respectively. Whereas John A. Jane [27] and Robert M. Starke [28] reported that GH hormonal remission rates were 33.3% (5/15) and 45% (9/20) in the binostril approach for the same grade tumors, respectively. For tumors with Hardy grade 3 and 4, the GH hormonal remission rates were 33.3% (5/15) in Gondim, J.A et al’s mononostril approach [23] and 52.1% (25/48) in Derya Brcu Hazer et al’s binostril approach [29].

The above literature showed that the binostril group seemed to have a tendency towards better outcomes for invasive macroadenomas pituitary adenomas. Unfortunately, subgroup analysis of the outcome based on Hardy grades and/or Knosp grades was not attained in our analysis because of the lack of suitable data. The Hardy grades and Knosp grades of the tumors was not recorded in most reports. When grades were reported, it was infrequently correlated to postoperative outcomes.

### Nasal complications

Since the binostril approach crosses the nasal septum, it results in greater surgical trauma to the nasal cavity. This meta-analysis revealed that the binostril group had a higher rate of epistaxis (1.5% versus 0.4%, p<0.05) than mononostril group. The binostril approach placed the posterior nasal branch of the sphenopalatine artery [27] and some nasal septum nerves at a greater risk than the mononostril approach. Therefore, the risks of epistaxis, olfactory damage, and postoperative discomfort are possibly increased. For olfactory damage, many studies within the binostril group reported some relevant data, for example, Charalampaki et al reported hyposmia was found in 10% of cases and anosmia was found in 2% [30]. Alterations in taste or smell was found in25.7% of cases and 30%was found in Starke et al´s [28] and John A. Jane et al´s [27] report, respectively. Dallapiazza et al reported that alteration in smell was found in 13% of cases [26]. Through above systematic review, we could found that the olfactory damage was more common in the binostril group. However, the number of studies relevant to olfactory damage was rare in the mononostril group; we postulate that olfactory damage was not a common complication of the mononostril group.

We also found the incidence of sinusitis is similar between these two approaches. This was most likely because sinusitis was related to many factors, such as materials of sellar floor reconstruction, antibiotic therapy, clearance of blood clots, and post operation care. We attempted to conduct a more detailed analysis of nasal complications, such as septal perforations, mucosal adhesions, and other sinonasal complaints. Unfortunately, we were unable to perform adequate comparisons with the available data, especially because of the lack data within the mononostril approach.

### Other parameters

Additionally, we discovered that the length of hospital stay in binostril group was shorter (3.2 days versus 4.4 days, p = 0.000) than that in the mononostril group. According to our experience, postoperative complications is an influential factor of increasing the length of hospital stay, such as postoperative CSF leaks and diabetes insipidus. The higher rate of anterior pituitary insufficiency and temporary diabetes insipidus we found in the monostril group would increase hospitalization days. Another important factor was the standard of discharge was not same in different medical center. Nevertheless, the exact cause for such result requires further research.

Endoscopic endonasal pituitary surgery is rapidly developing in surgical discipline. With its main targets being more minimally invasive and better results. We believe that this meta-analysis affords new reference evident to the choice of mononostril or binostril endoscopic endonasal transsphenoidal approaches through the inclusion of the vast amount of studies and patients in the analyses than previous reports. Finally, future comparative studies would provide stronger inferences and more credible data, such as meta-analyses with less bias, more rigorous subgroup analysis and multicenter comparisons with attention to surgical expertise, and volume.

### Limitations

Almost all included reports are single-armed, uncontrolled studies with an obvious bias for lack of a control group such as no measured publication bias. It is impossible to discern whether authors tend to publish results that appear more favorable from the literature. We were unable to obtain the available data recorded the actual size of the tumors, and we did not know whether the mean size of pituitary adenomas had a statistically significant difference between the two groups. Very few studies reported correlate outcomes with primary/revision surgery and some statistical results of this study were deficient in adequate data. We also had to recognize that this analysis represented only the results of early outcomes and complications, and there were rarely long-term studies following these patients beyond the initial postoperative period published.

## Conclusion

The results of this meta-analysis indicated that the binostril approach had less anterior pituitary insufficiency, less temporary diabetes insipidus, and shorter hospitalization days than the mononostril approach. Meanwhile, the binostril had higher rate of epistaxis. Additionally, the binostril group seems to suggest a tendency towards better outcomes for invasive macroadenomas.

## Supporting Information

S1 Checklist PRISMA Checklist. (DOC)

S1 Fig A detailed flowchar of the included studies. (TIF)

S2 Fig Tumor resection rate (GTR) in mononostril (mon): 77.8%. (TIF)

S3 Fig GTR in binostril (bi): 80.3%. (TIF)

S4 Fig GTR of pituitary macroadenomas in mon: 70.4%. (TIF)

S5 Fig GTR of pituitary macroadenomas in bi: 72.7%. (TIF)

S6 Fig Hormonal remission rate in mon: 72.9%. (TIF)

S7 Fig Hormonal remission rate in bi: 76.0%. (TIF)

S8 Fig GH remission rate in mon: 73.4%. (TIF)

S9 Fig GH remission rate in bi: 75.5%. (TIF)

S10 Fig PRL remission rate in mon: 70.9%. (TIF)

S11 Fig PRL remission rate in bi: 73.1%. (TIF)

S12 Fig ACTH remission rate in mon: 80.3%. (TIF)

S13 Fig ACTH remission rate in bi: 80.9%. (TIF)

S14 Fig Improvement in visual function in mon: 91.1. (TIF)

S15 Fig Improvement in visual function in bi: 88.0%. (TIF)

S16 Fig CSF leakage in mon: 2.9%. (TIF)

S17 Fig CSF leakage in bi: 3.1%. (TIF)

S18 Fig Temporary diabetes insipidus in mon: 5.3%. (TIF)

S19 Fig Temporary diabetes insipidus in bi: 2.9%. (TIF)

S20 Fig Permanent diabetes insipidus in mon: 1.0%. (TIF)

S21 Fig Permanent diabetes insipidus in bi: 0.9%. (TIF)

S22 Fig Anterior pituitary insufficiency in mon: 6.4%. (TIF)

S23 Fig Anterior pituitary insufficiency in bi: 2.3%. (TIF)

S24 Fig Meningitis in mon: 0.8%. (TIF)

S25 Fig Meningitis in bi: 0.8%. (TIF)

S26 Fig Sinusitis in mon: 0.3%. (TIF)

S27 Fig Sinusitis in bi: 0.9%. (TIF)

S28 Fig Epistaxis in bi: 0.4%. (TIF)

S29 Fig Epistaxis in bi: 1.5%. (TIF)

S1 File The key phrases for searching from databases. (DOC)

S1 Table The characteristic of the included studies. (DOC)

S2 Table Patient characteristics. (DOC)

S3 Table The length of hospital stay. (DOC)
