# Supplementary figures and images for: Mononostril versus Binostril Endoscopic Transsphenoidal Approach for Pituitary Adenomas: A Systematic Review and Meta-Analysis
Source: PLoS One. 2016 Apr 28;11(4):e0153397. doi: 10.1371/journal.pone.0153397 (PMC4849742; doi:10.1371/journal.pone.0153397)

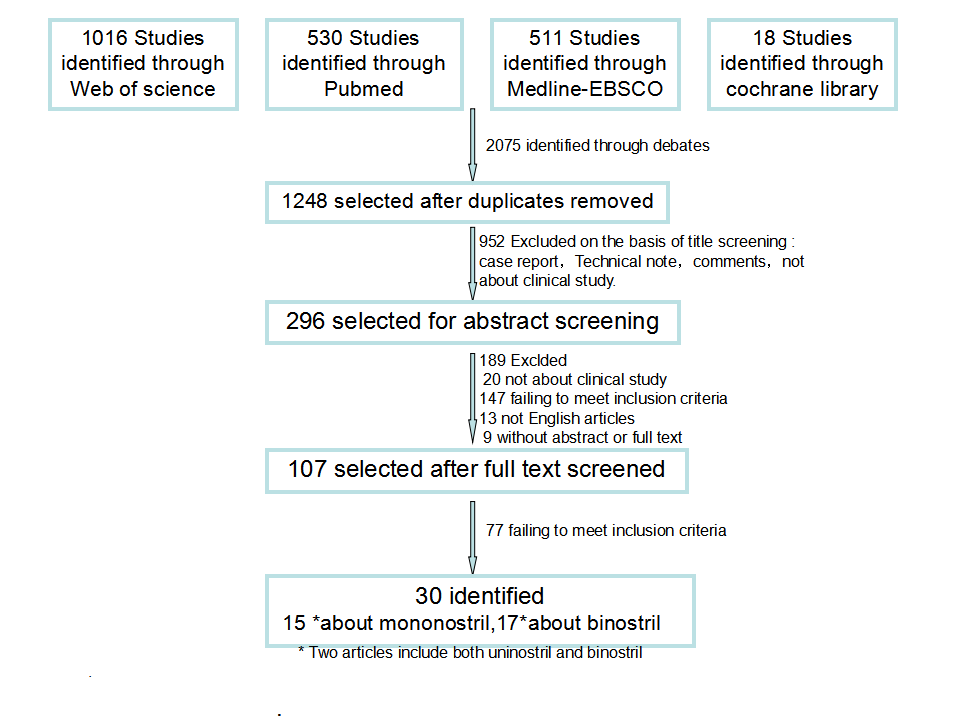

Supplement: S1 Fig — (TIF) [file pone.0153397.s002.tif]

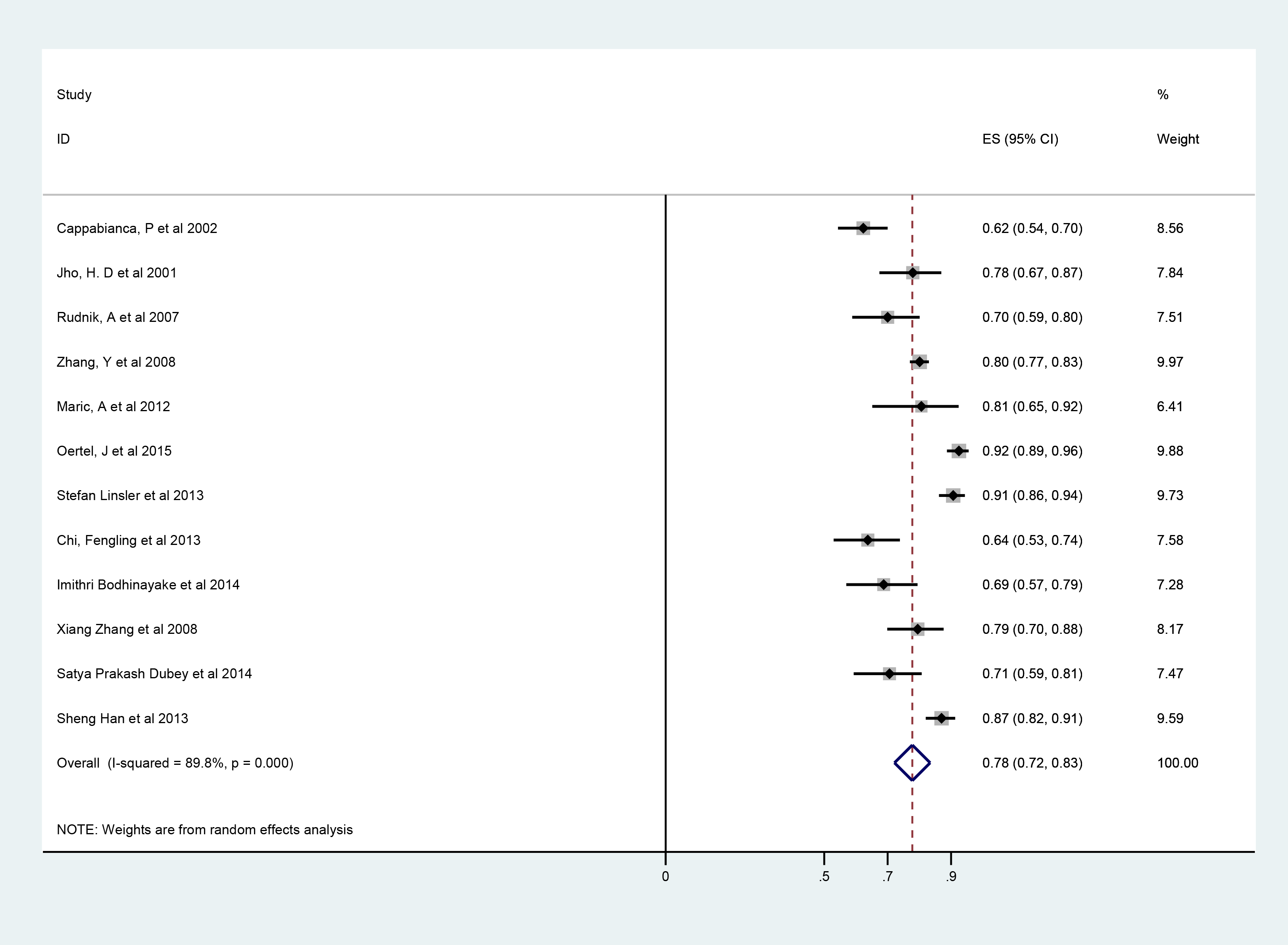

Supplement: S2 Fig — (TIF) [file pone.0153397.s003.tif]

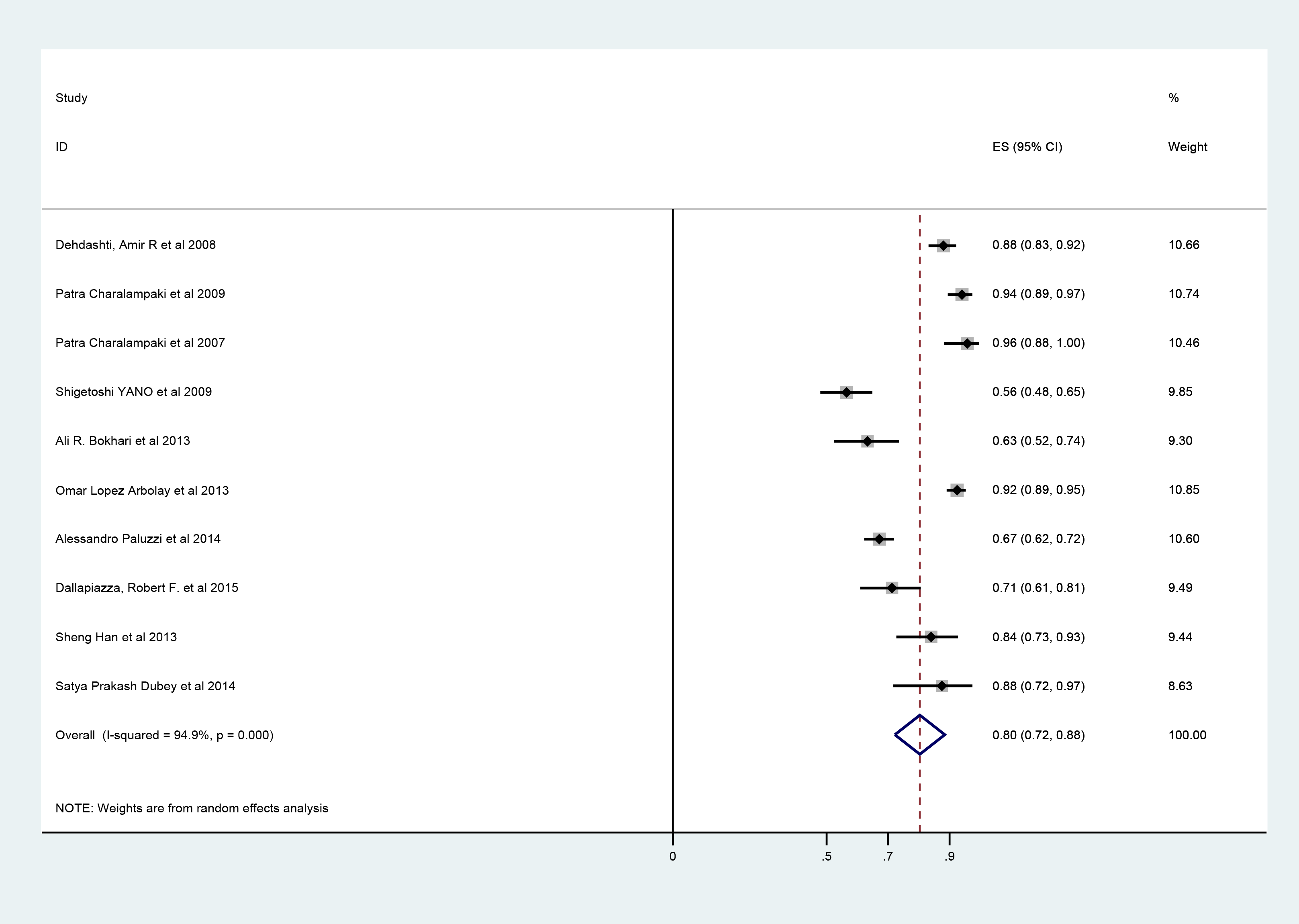

Supplement: S3 Fig — (TIF) [file pone.0153397.s004.tif]

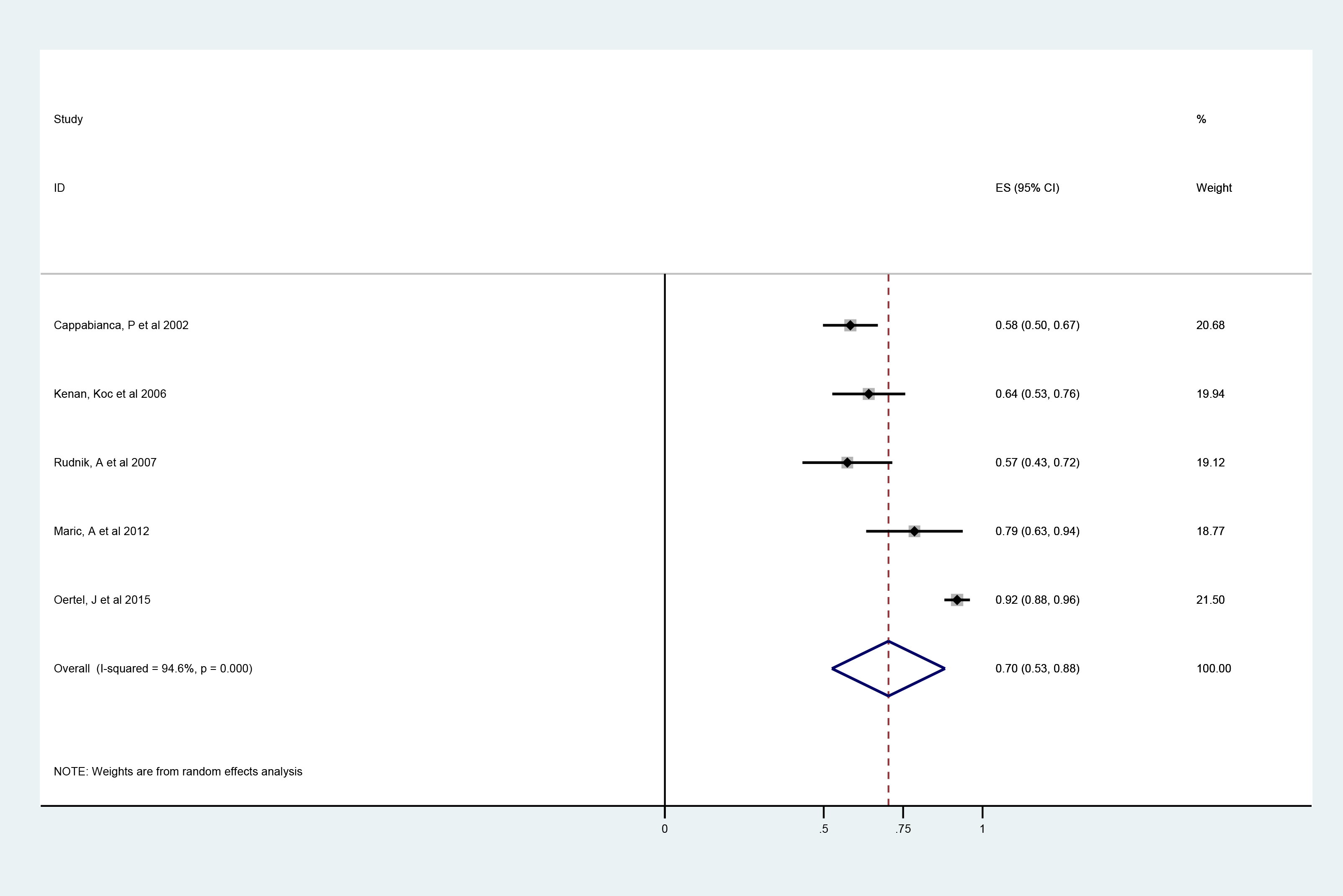

Supplement: S4 Fig — (TIF) [file pone.0153397.s005.tif]

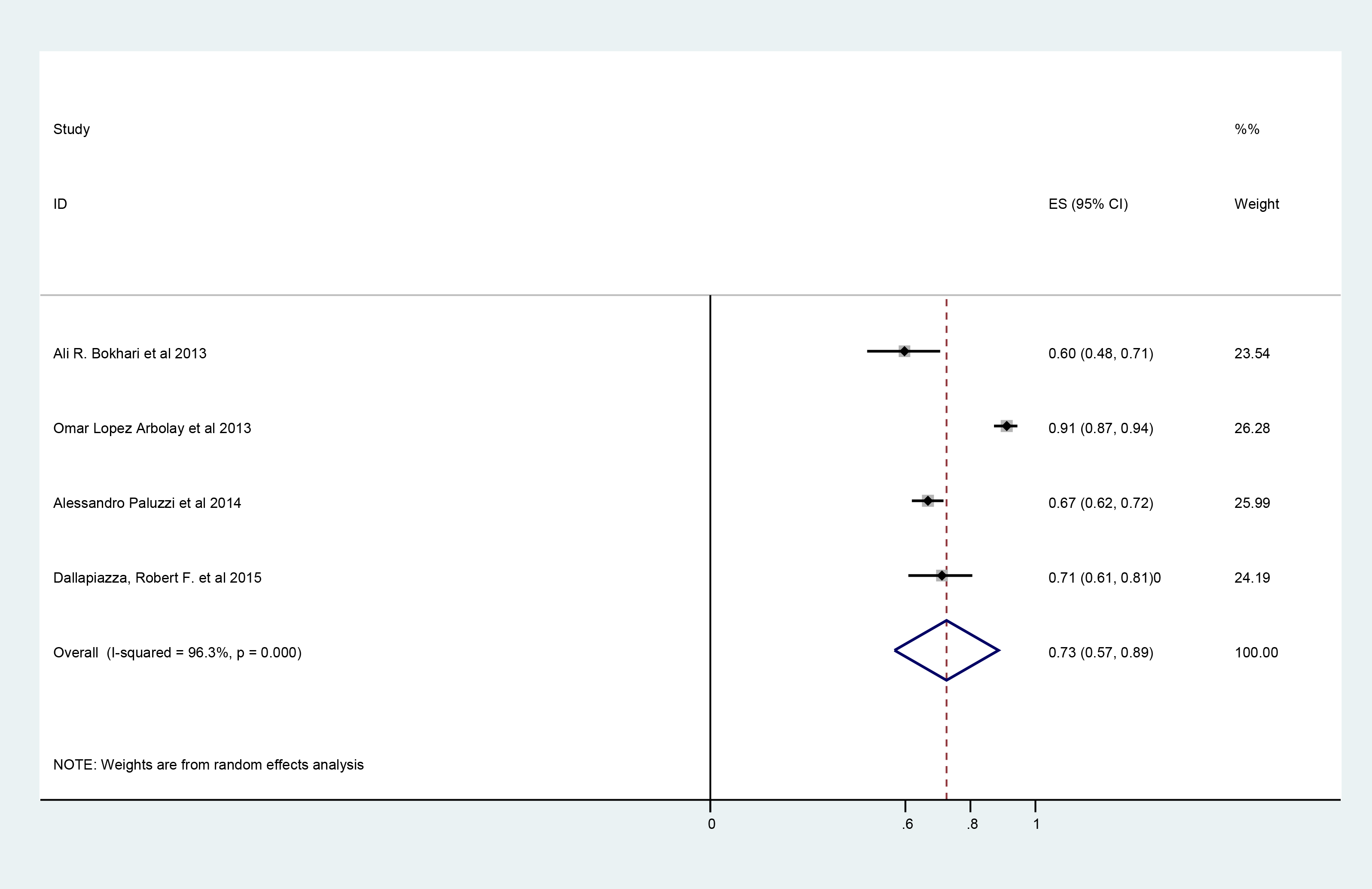

Supplement: S5 Fig — (TIF) [file pone.0153397.s006.tif]

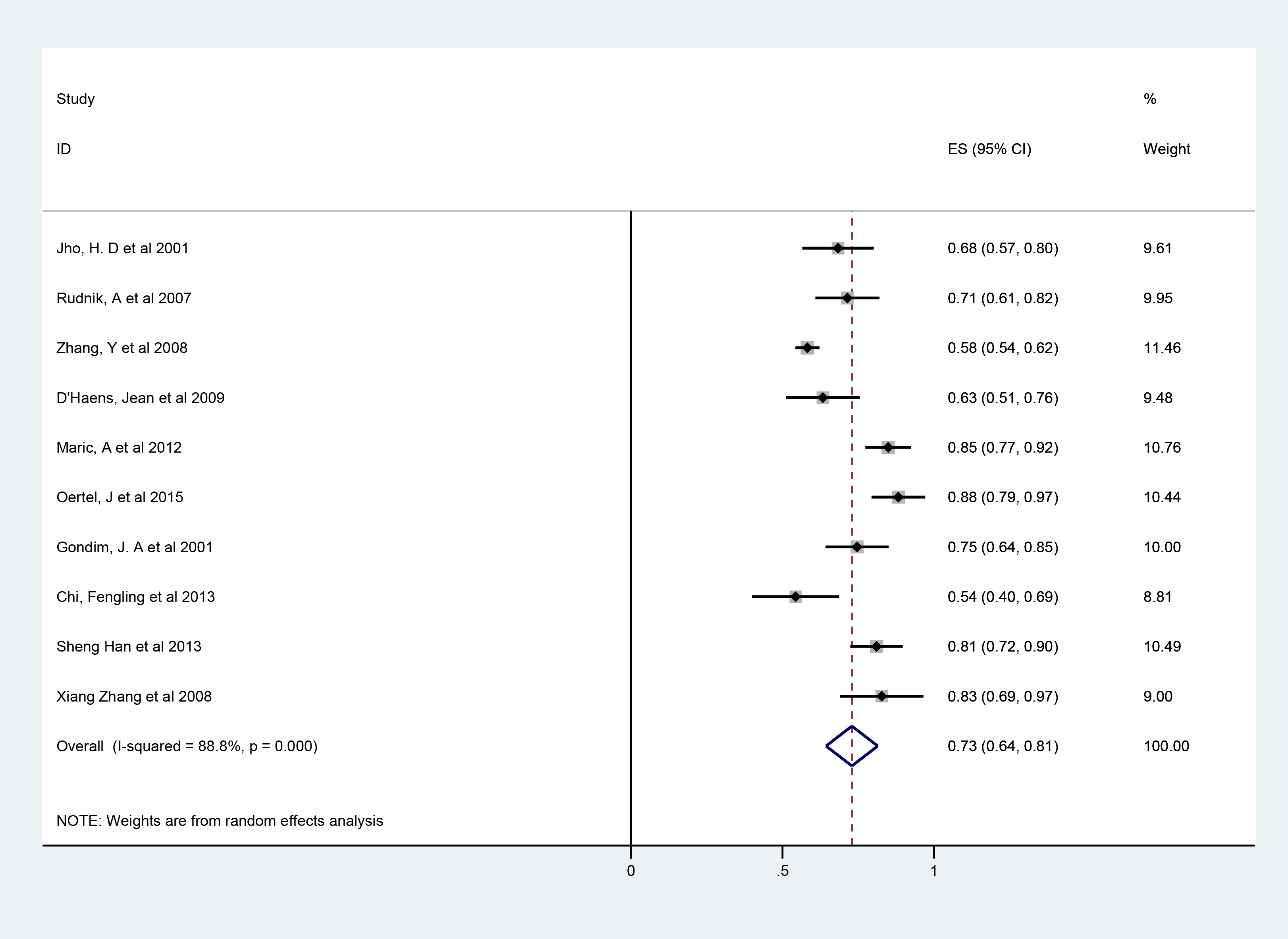

Supplement: S6 Fig — (TIF) [file pone.0153397.s007.tif]

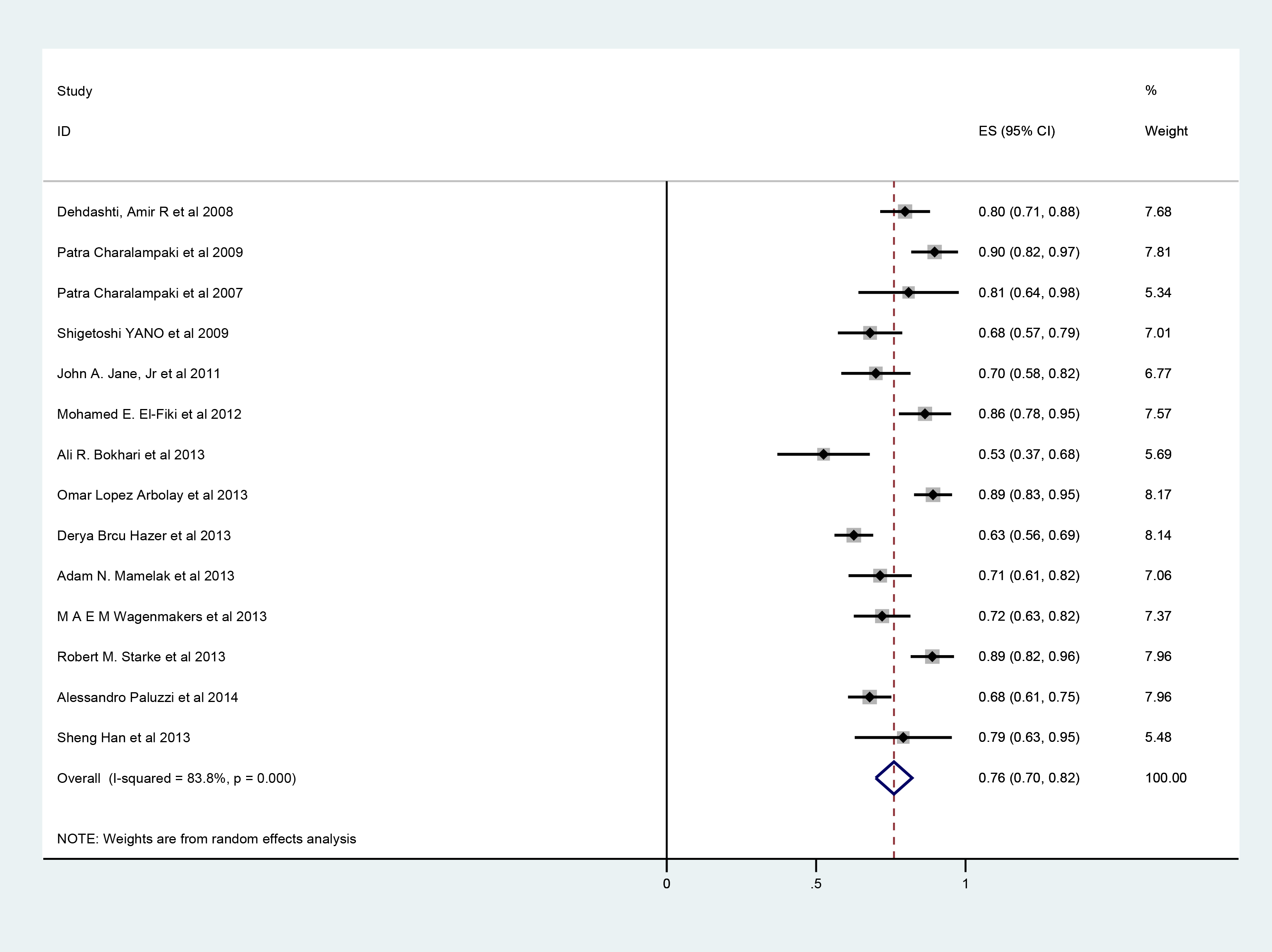

Supplement: S7 Fig — (TIF) [file pone.0153397.s008.tif]

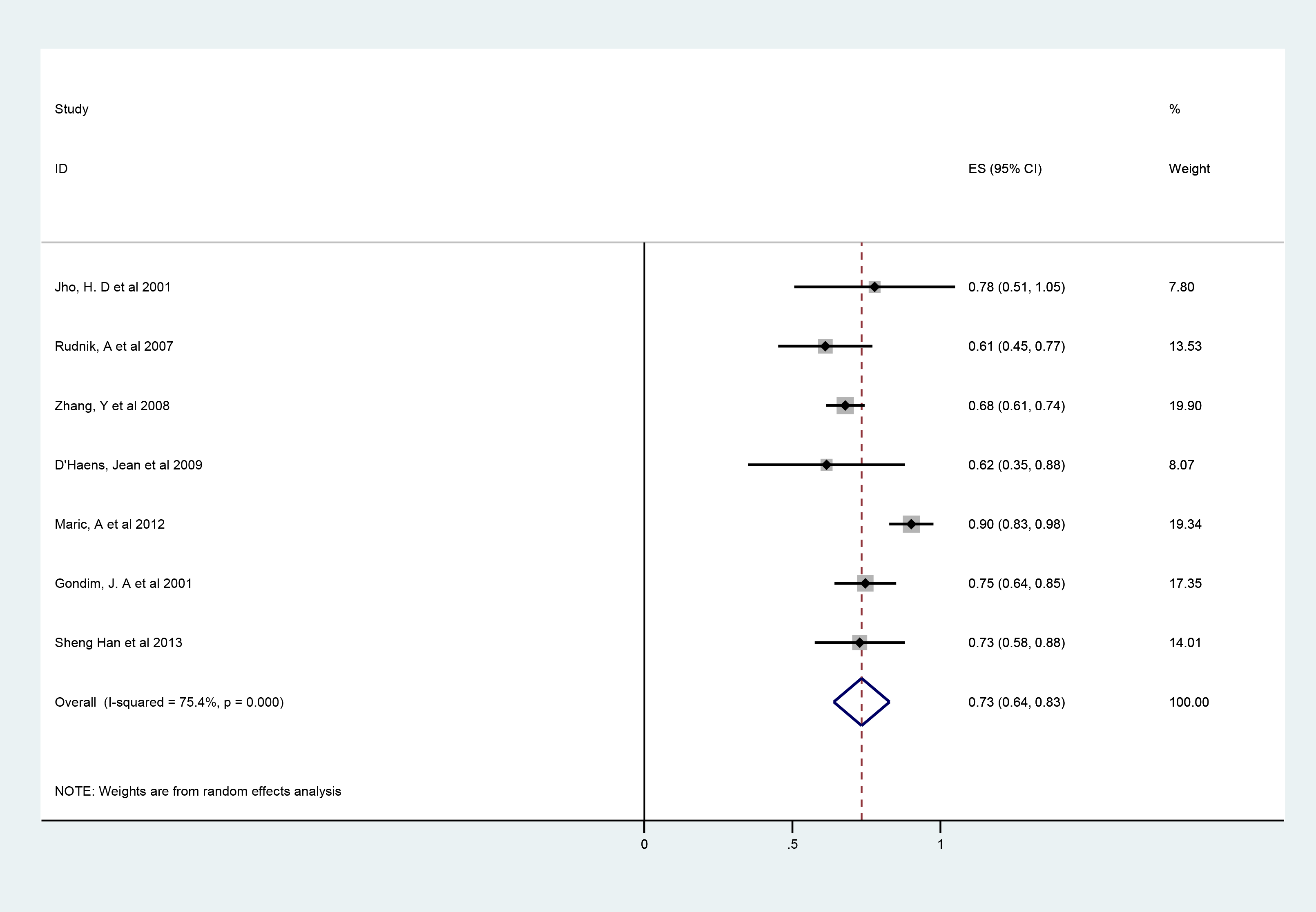

Supplement: S8 Fig — (TIF) [file pone.0153397.s009.tif]

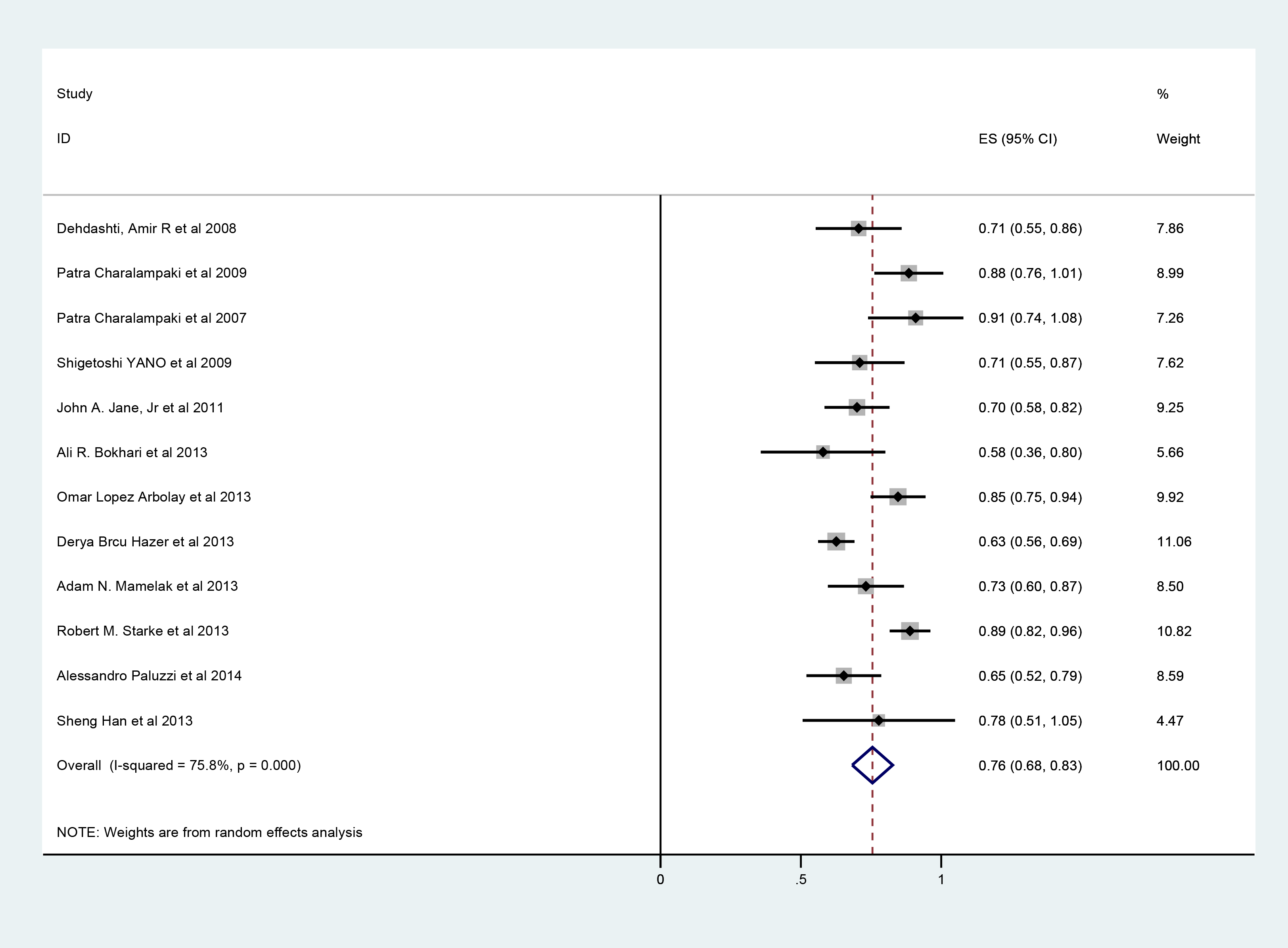

Supplement: S9 Fig — (TIF) [file pone.0153397.s010.tif]

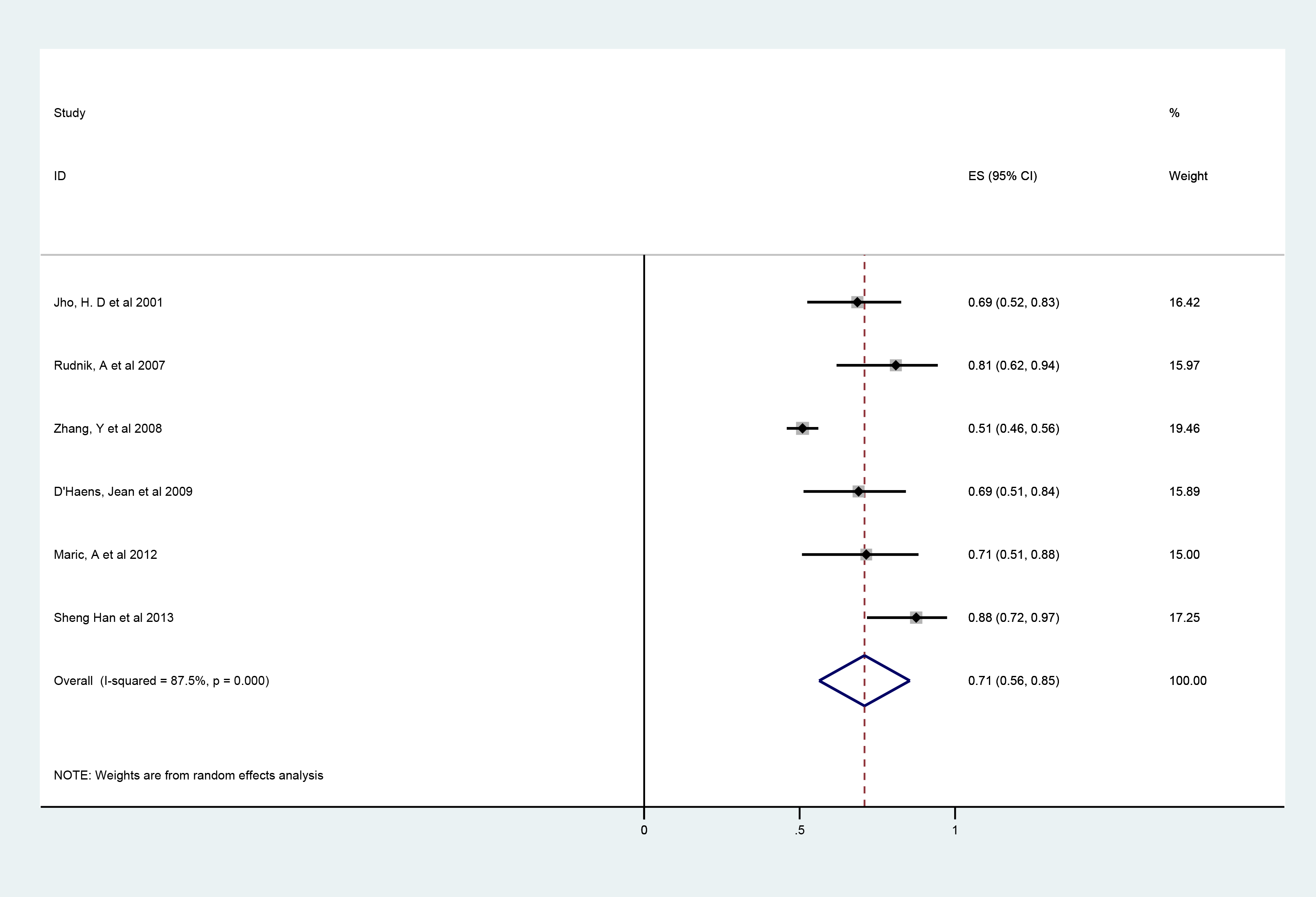

Supplement: S10 Fig — (TIF) [file pone.0153397.s011.tif]

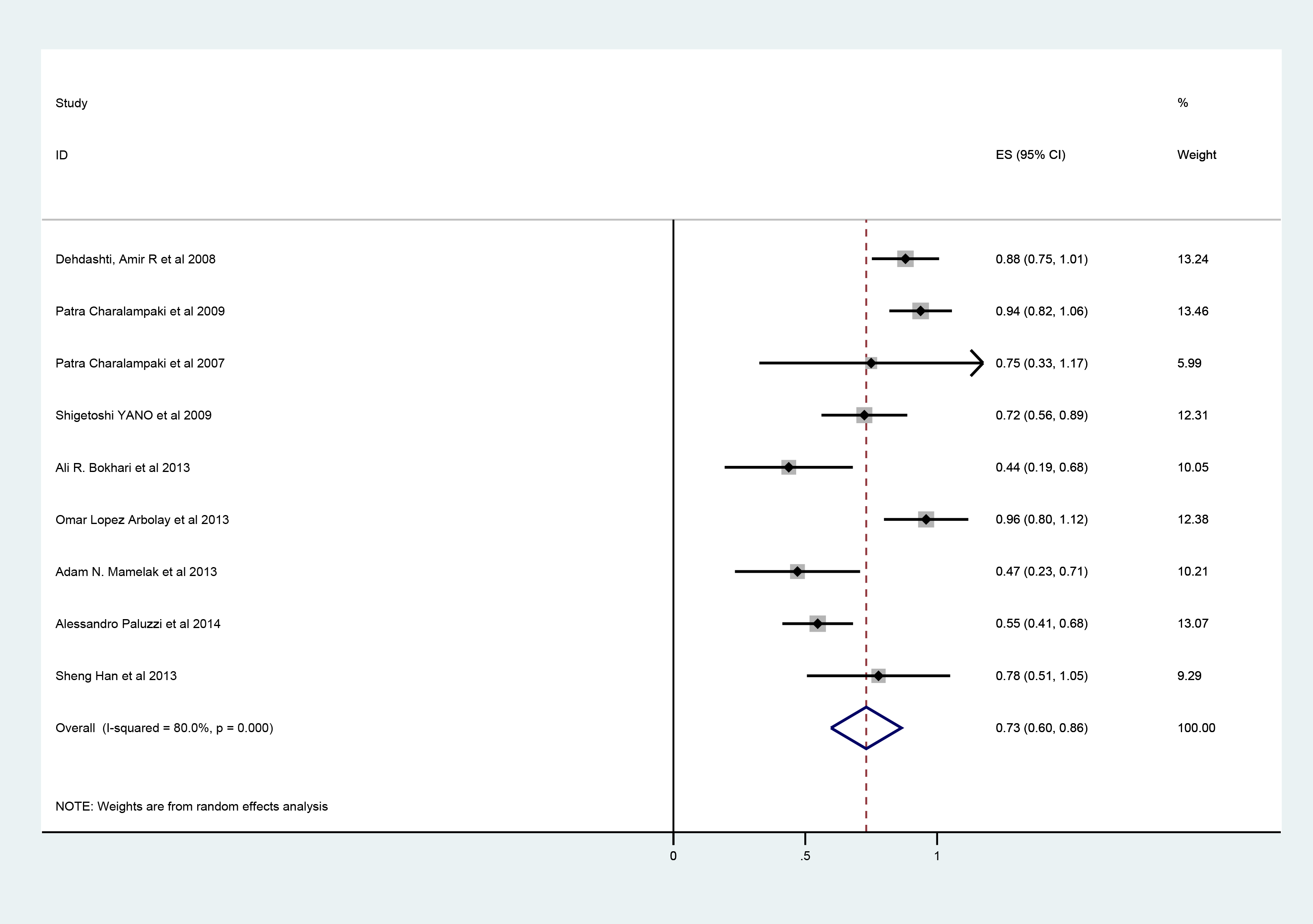

Supplement: S11 Fig — (TIF) [file pone.0153397.s012.tif]

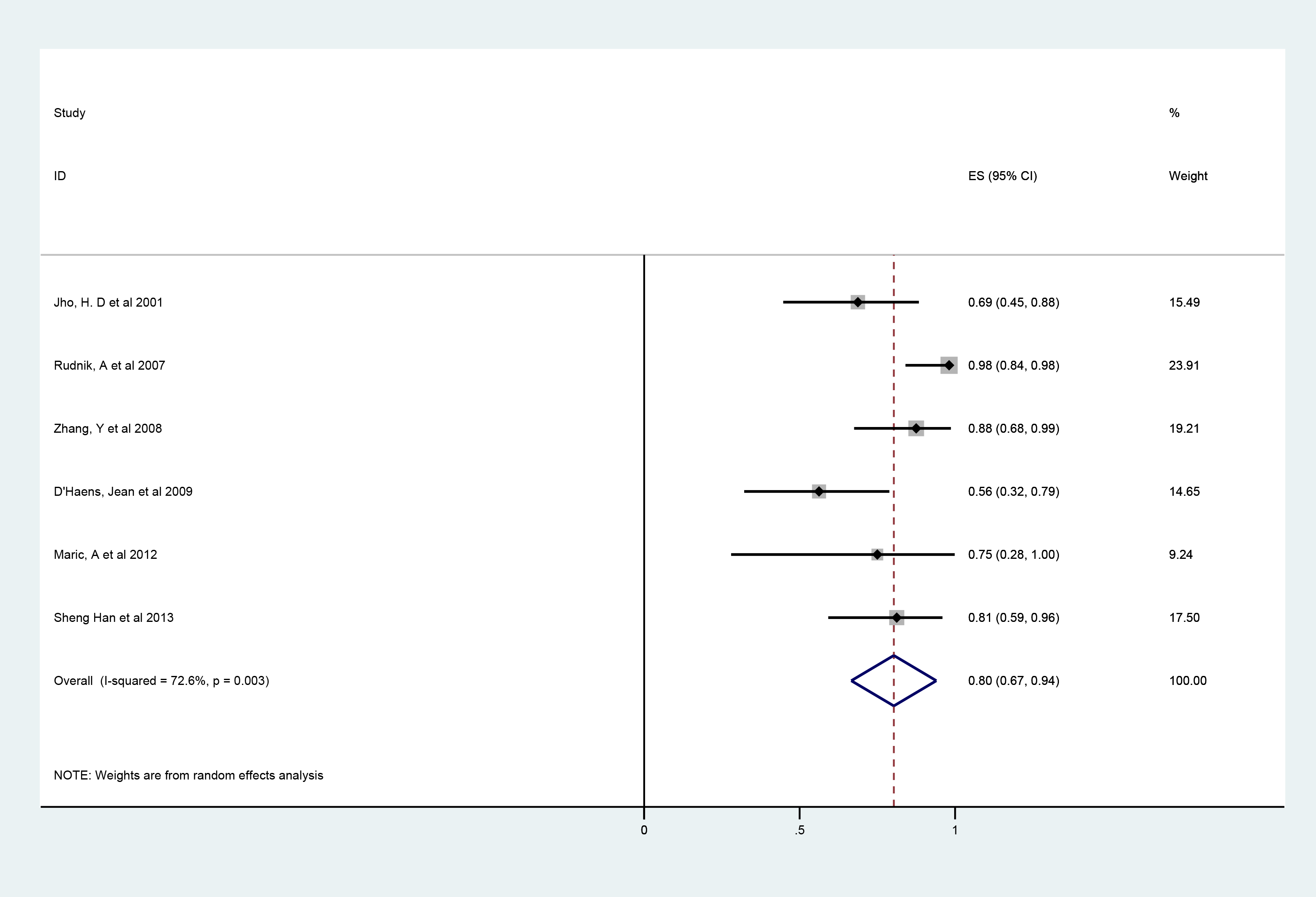

Supplement: S12 Fig — (TIF) [file pone.0153397.s013.tif]

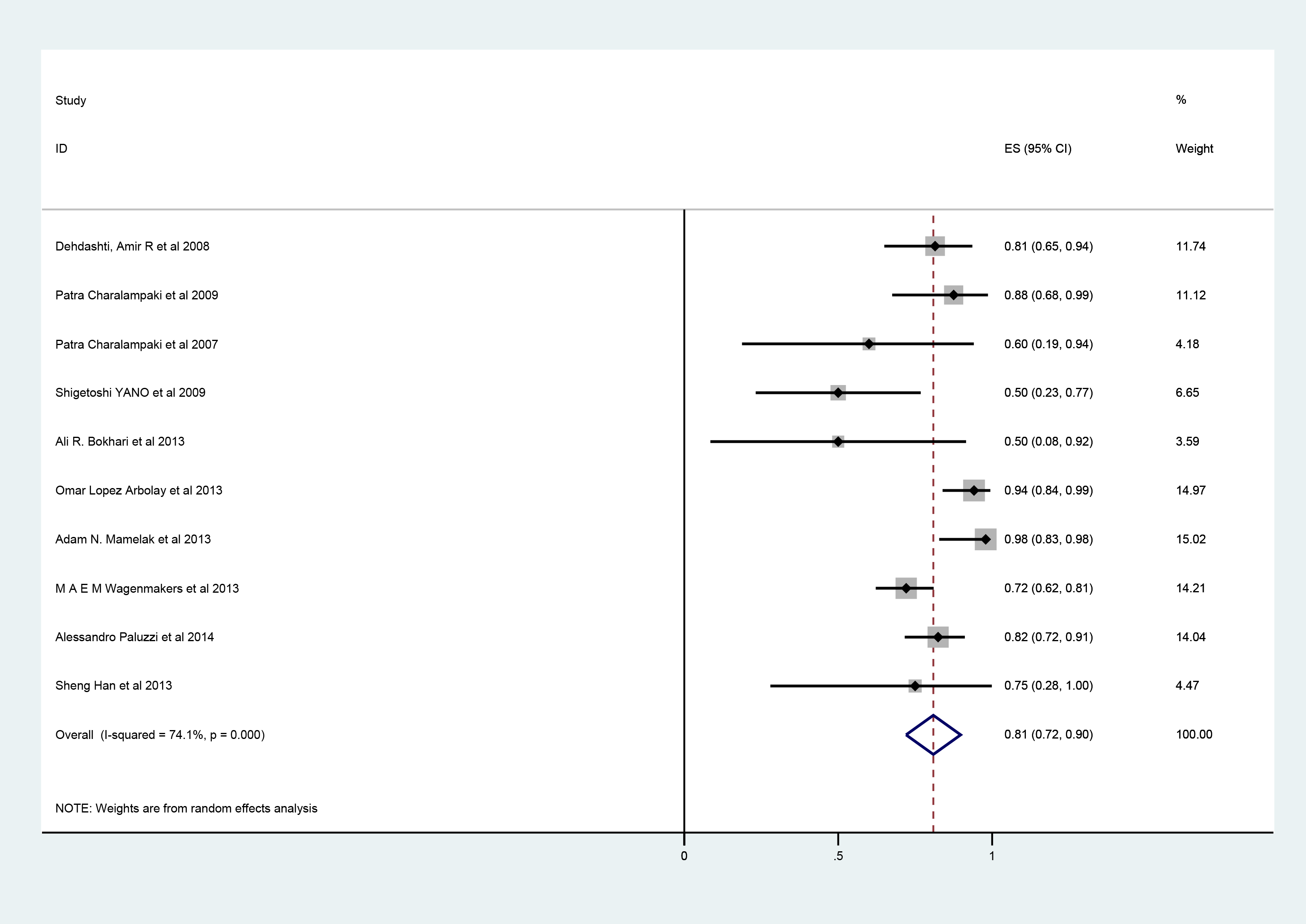

Supplement: S13 Fig — (TIF) [file pone.0153397.s014.tif]

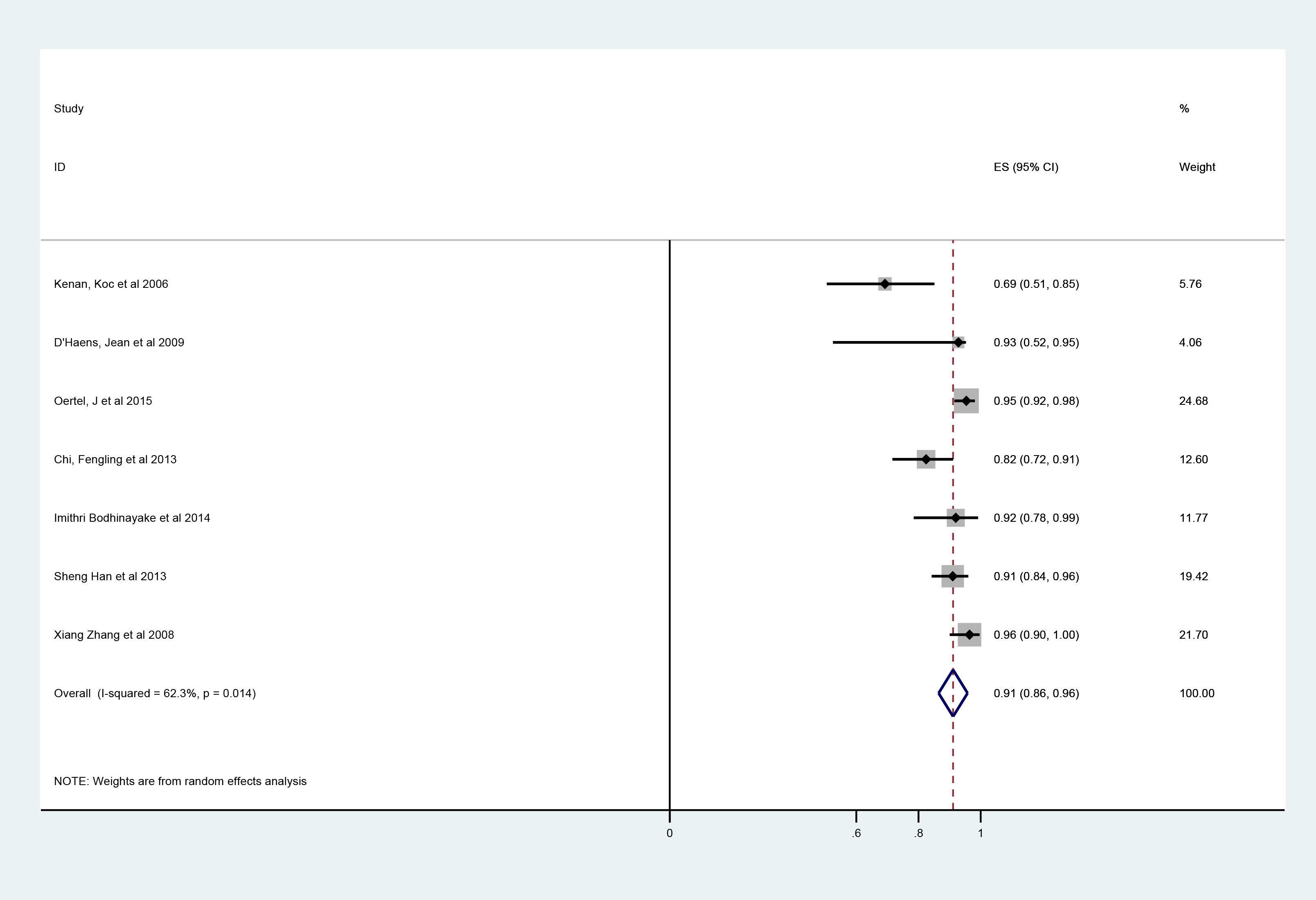

Supplement: S14 Fig — (TIF) [file pone.0153397.s015.tif]

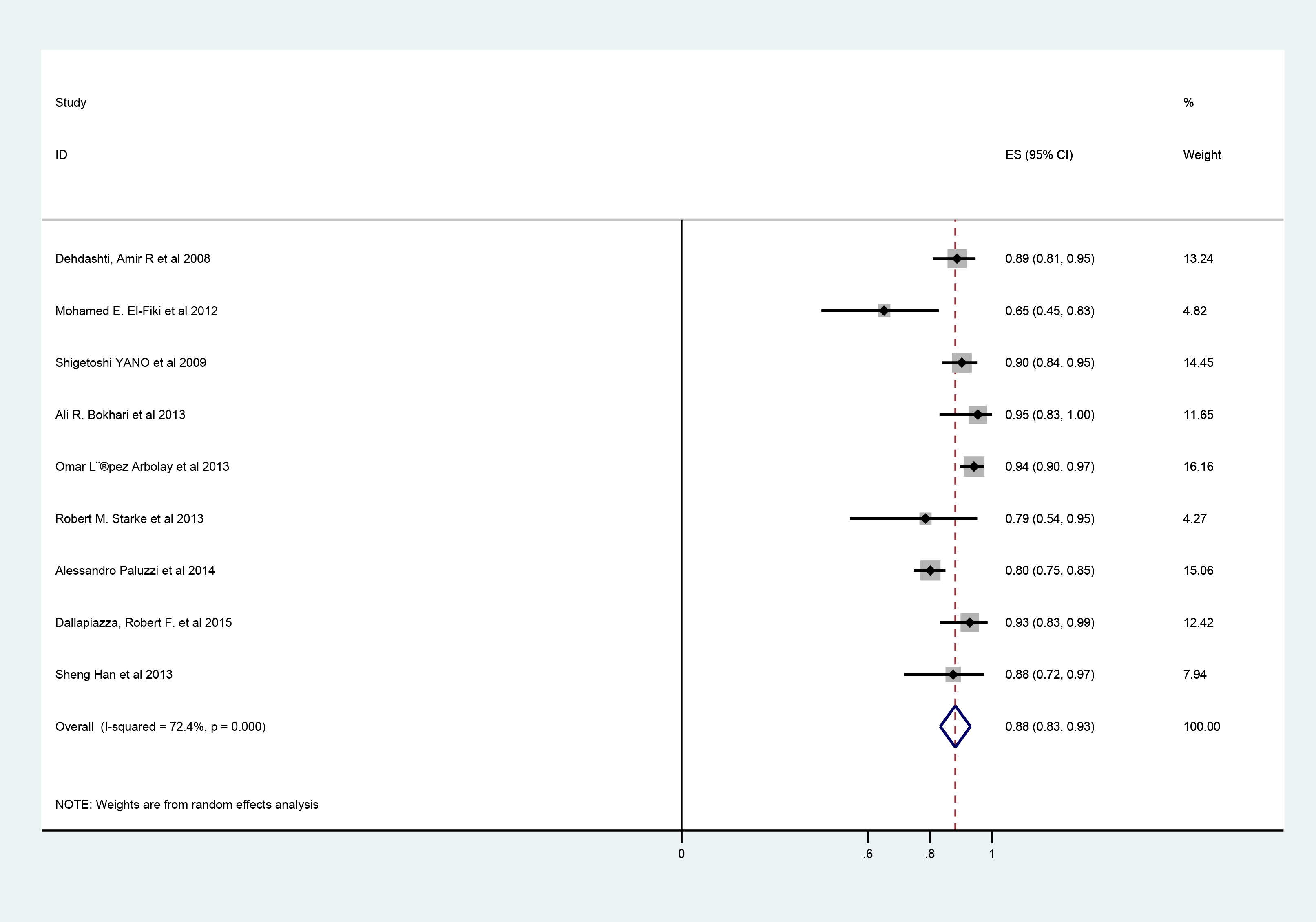

Supplement: S15 Fig — (TIF) [file pone.0153397.s016.tif]

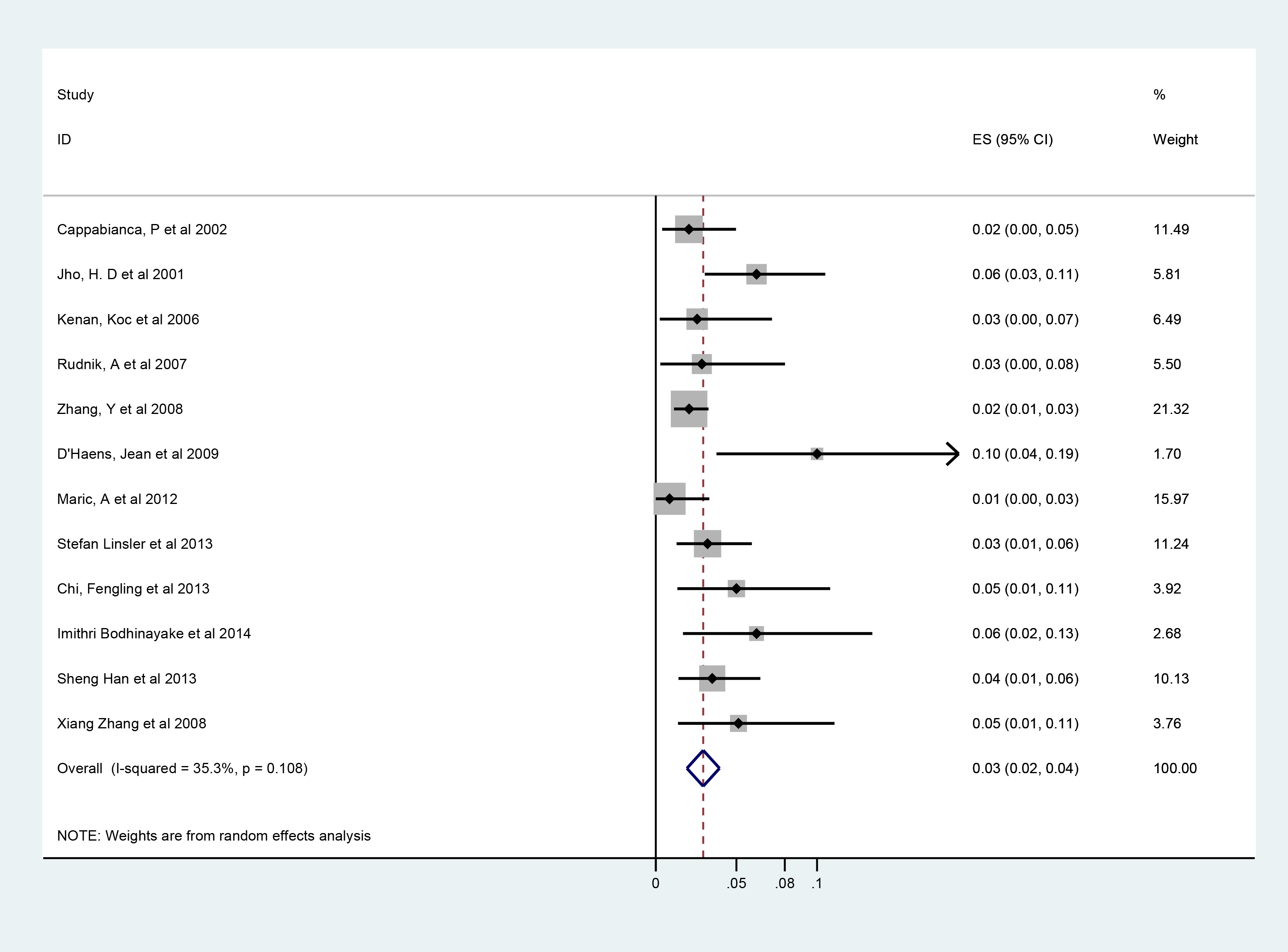

Supplement: S16 Fig — (TIF) [file pone.0153397.s017.tif]

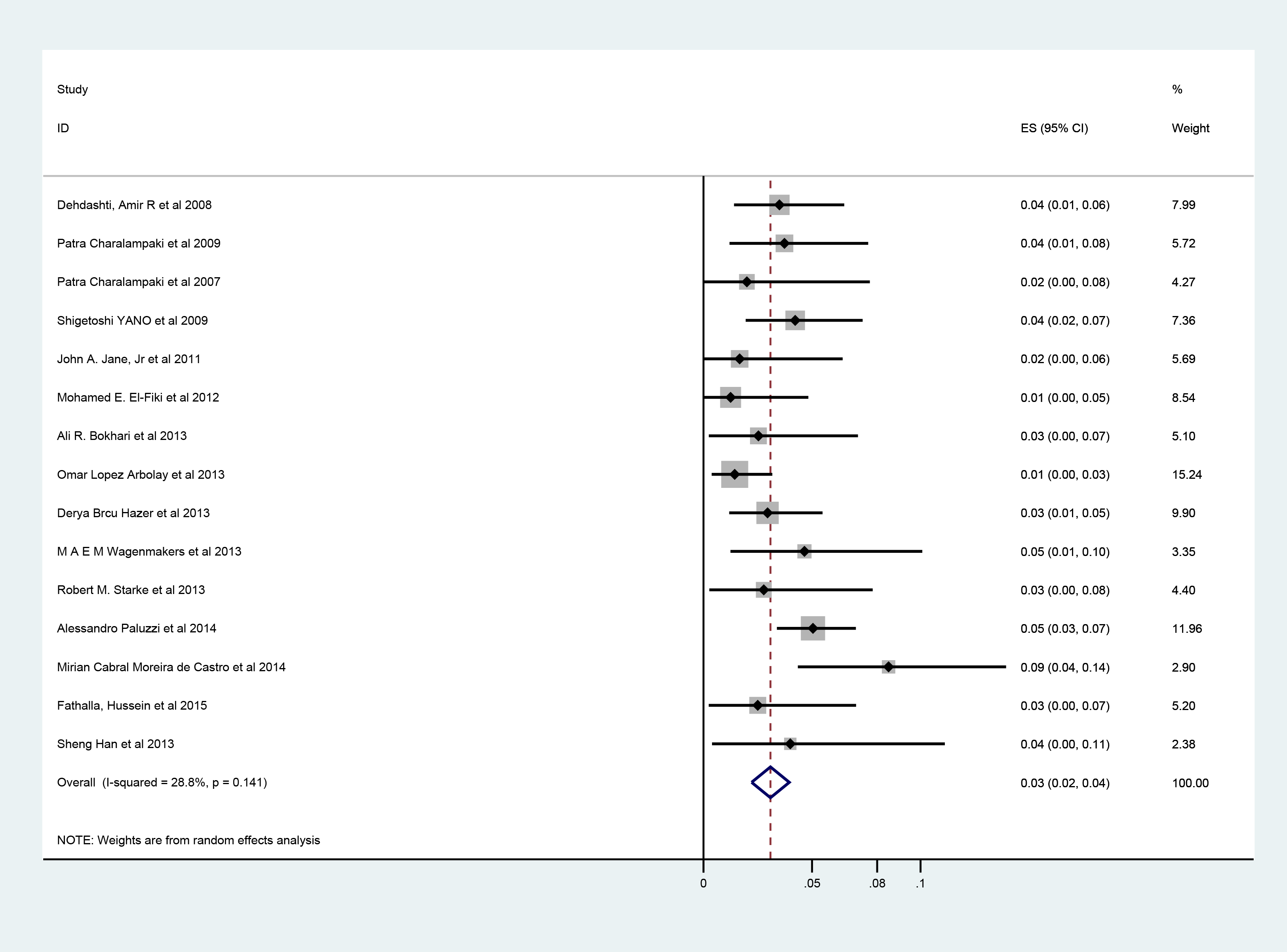

Supplement: S17 Fig — (TIF) [file pone.0153397.s018.tif]

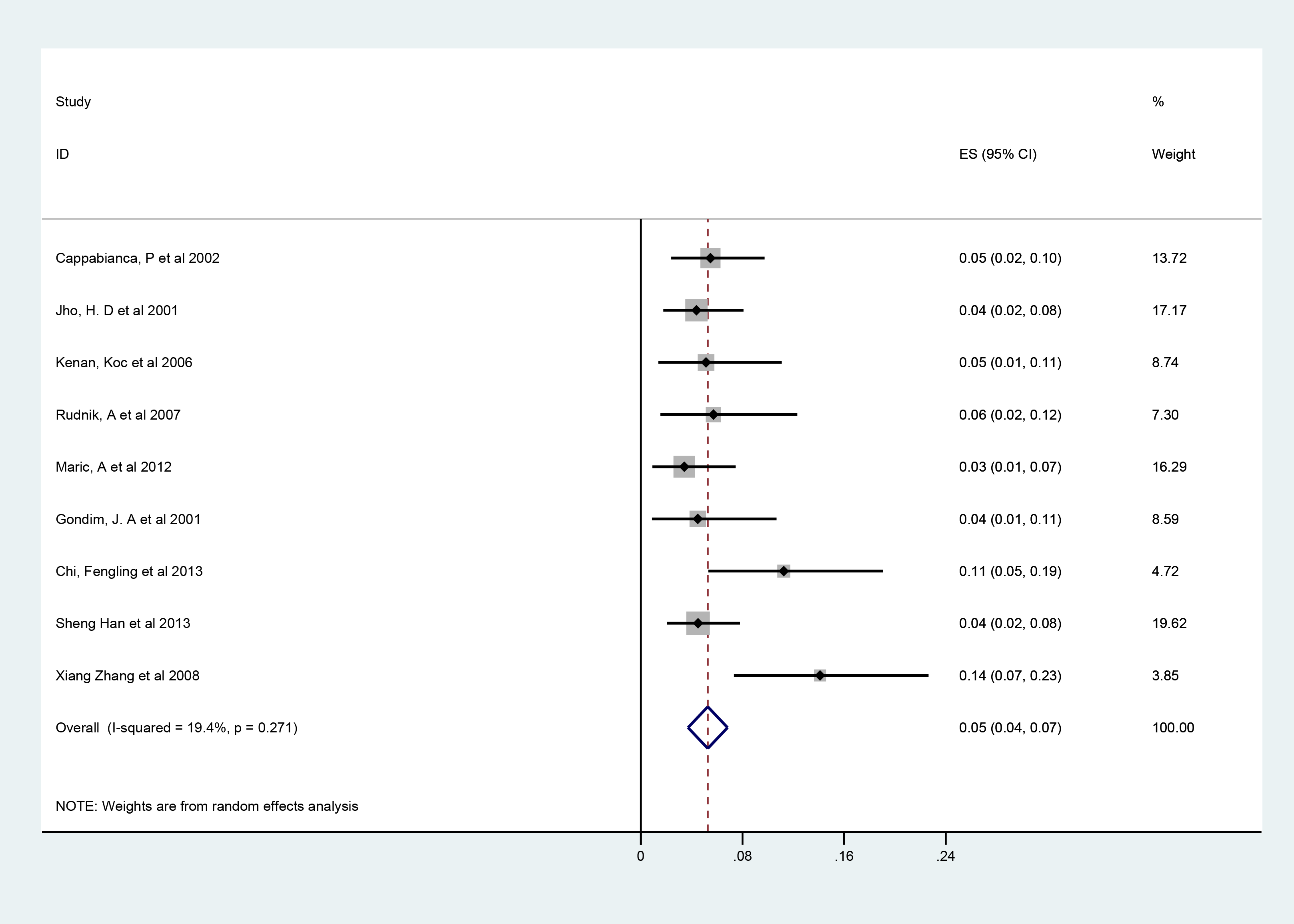

Supplement: S18 Fig — (TIF) [file pone.0153397.s019.tif]

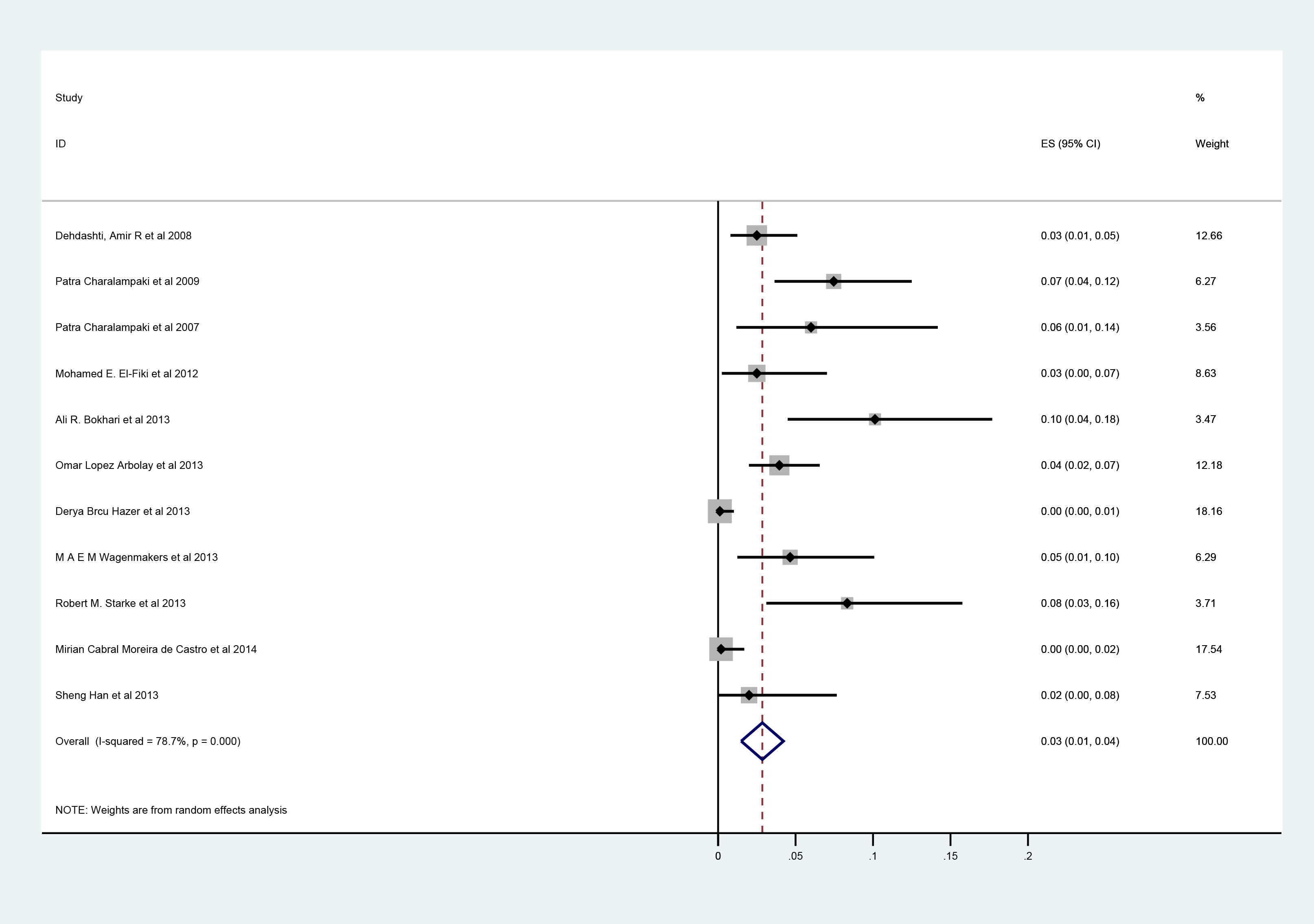

Supplement: S19 Fig — (TIF) [file pone.0153397.s020.tif]

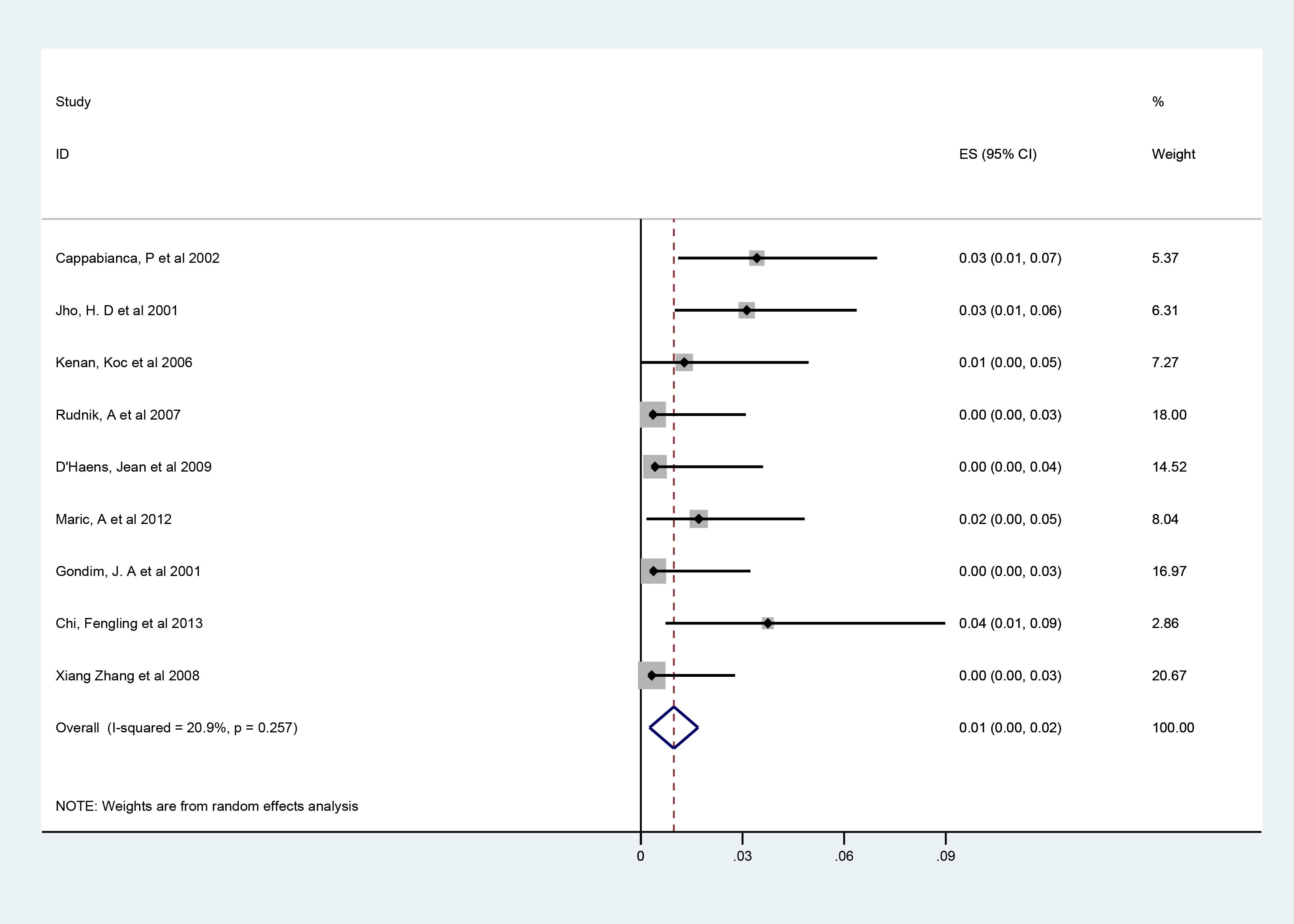

Supplement: S20 Fig — (TIF) [file pone.0153397.s021.tif]

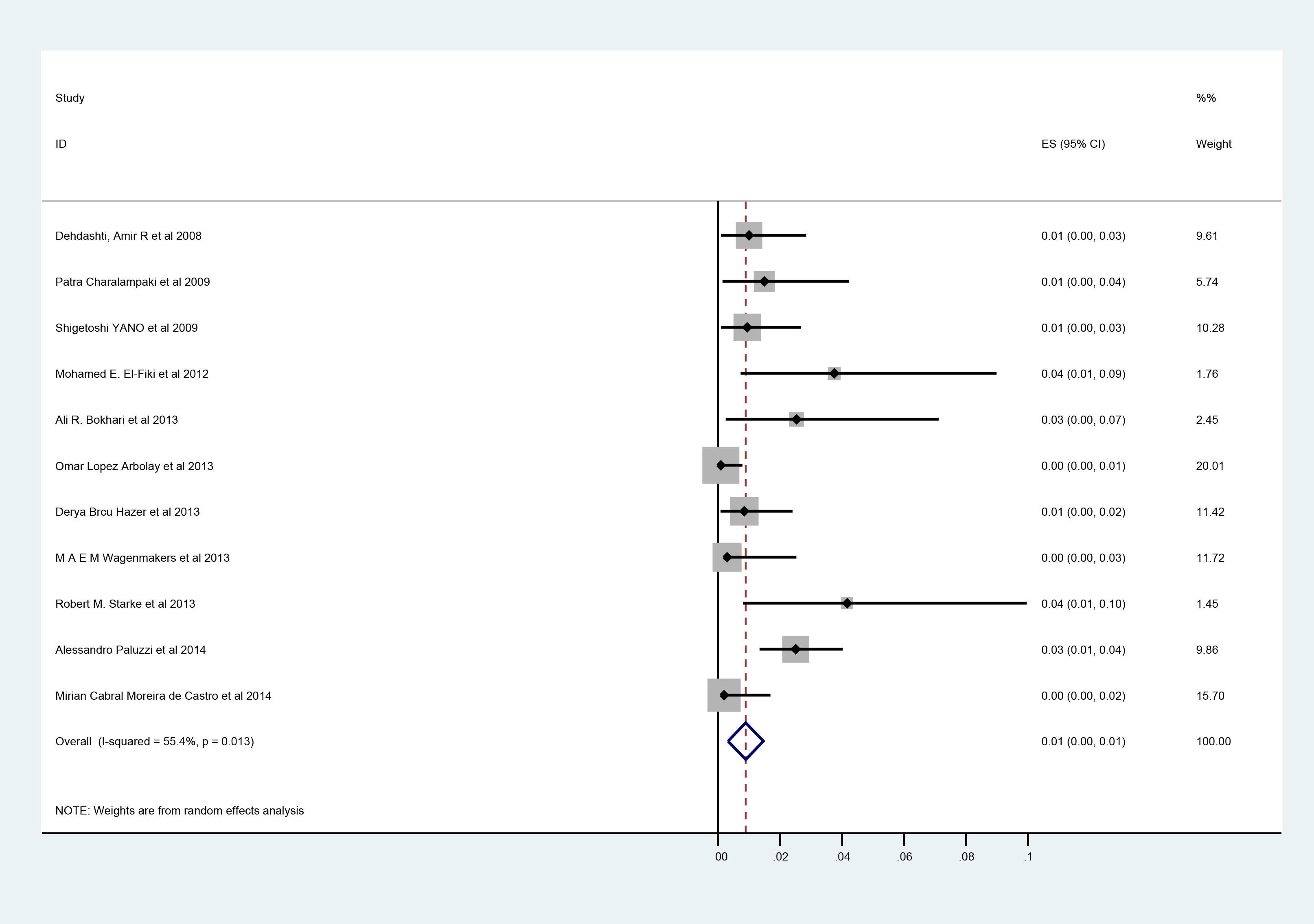

Supplement: S21 Fig — (TIF) [file pone.0153397.s022.tif]

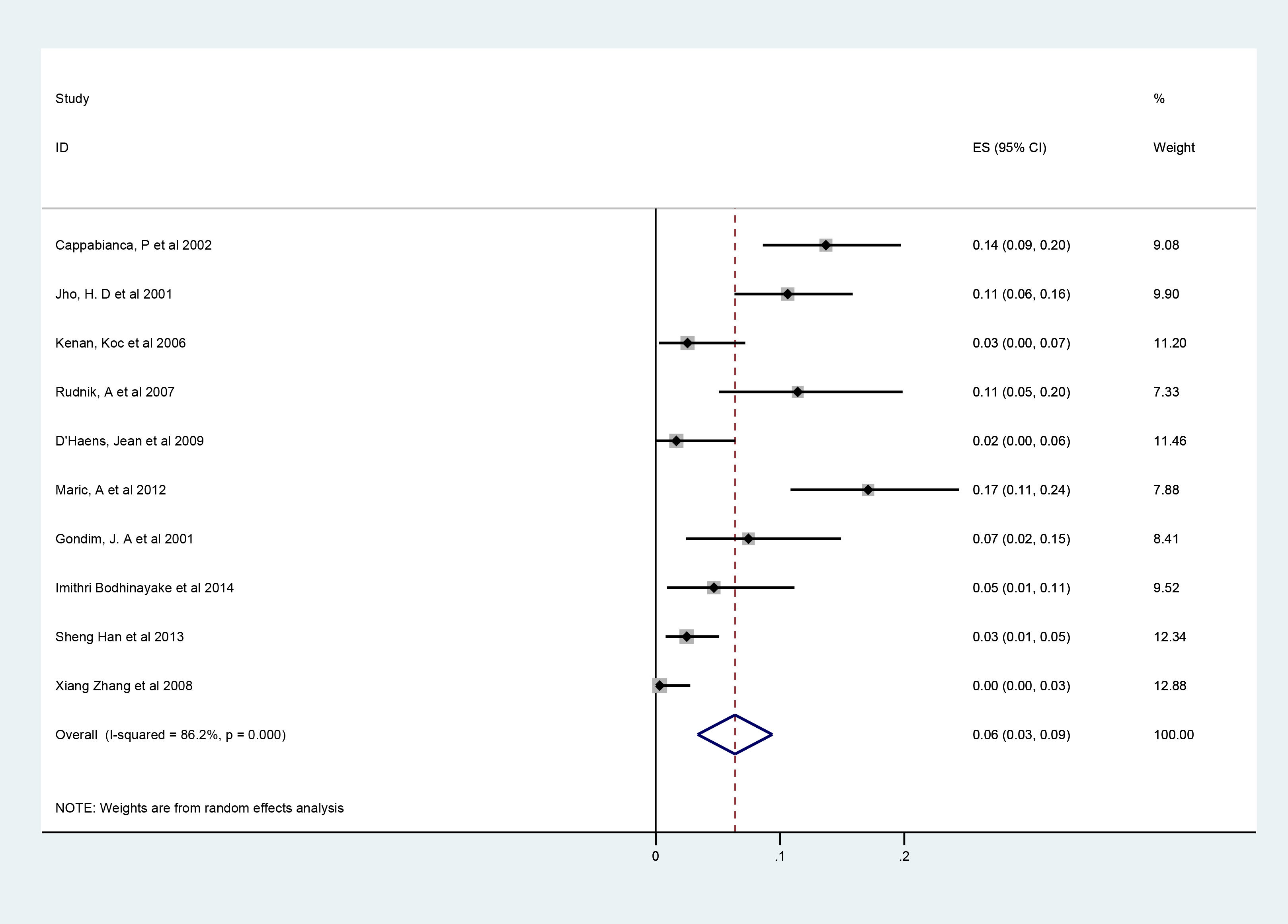

Supplement: S22 Fig — (TIF) [file pone.0153397.s023.tif]

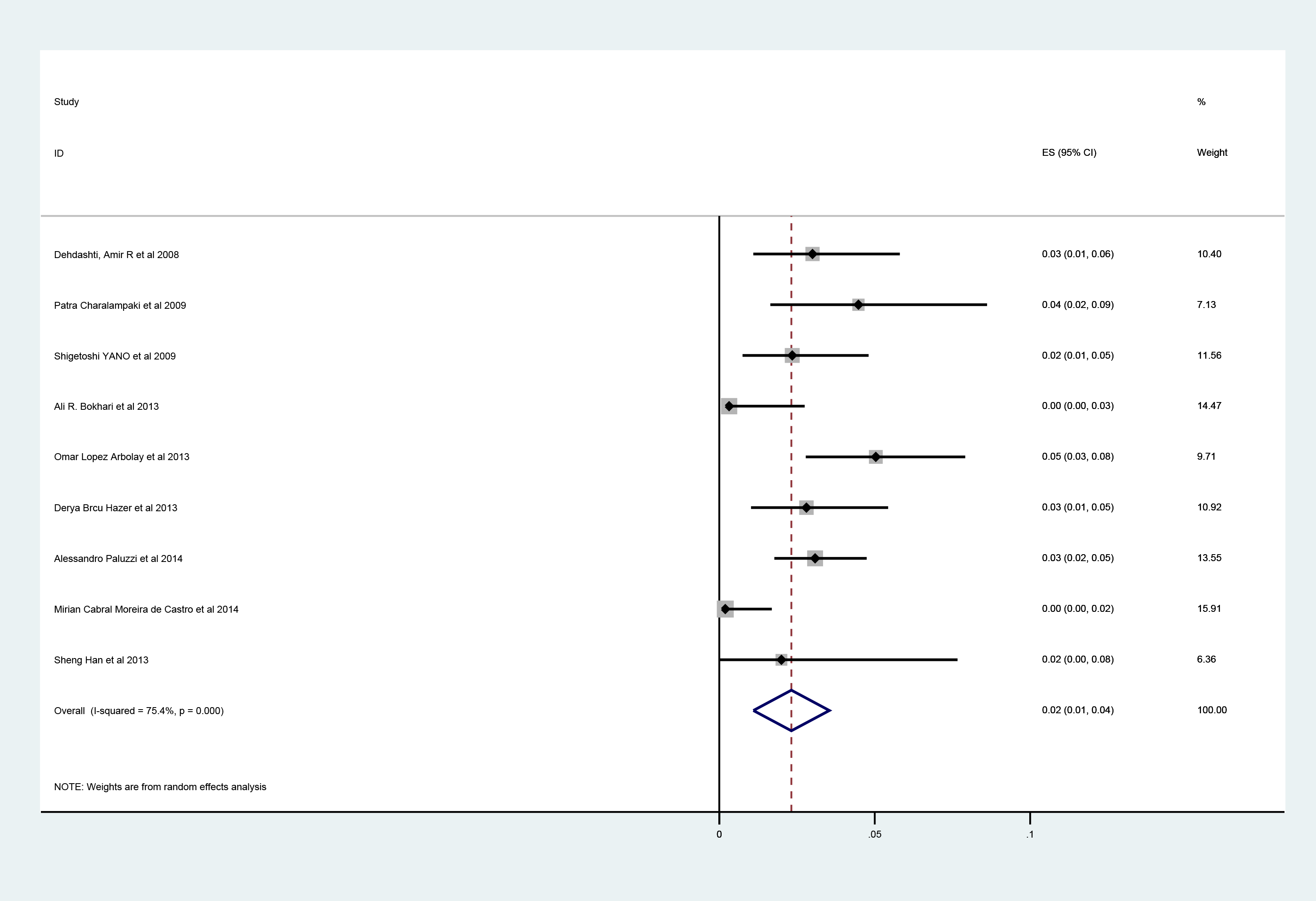

Supplement: S23 Fig — (TIF) [file pone.0153397.s024.tif]

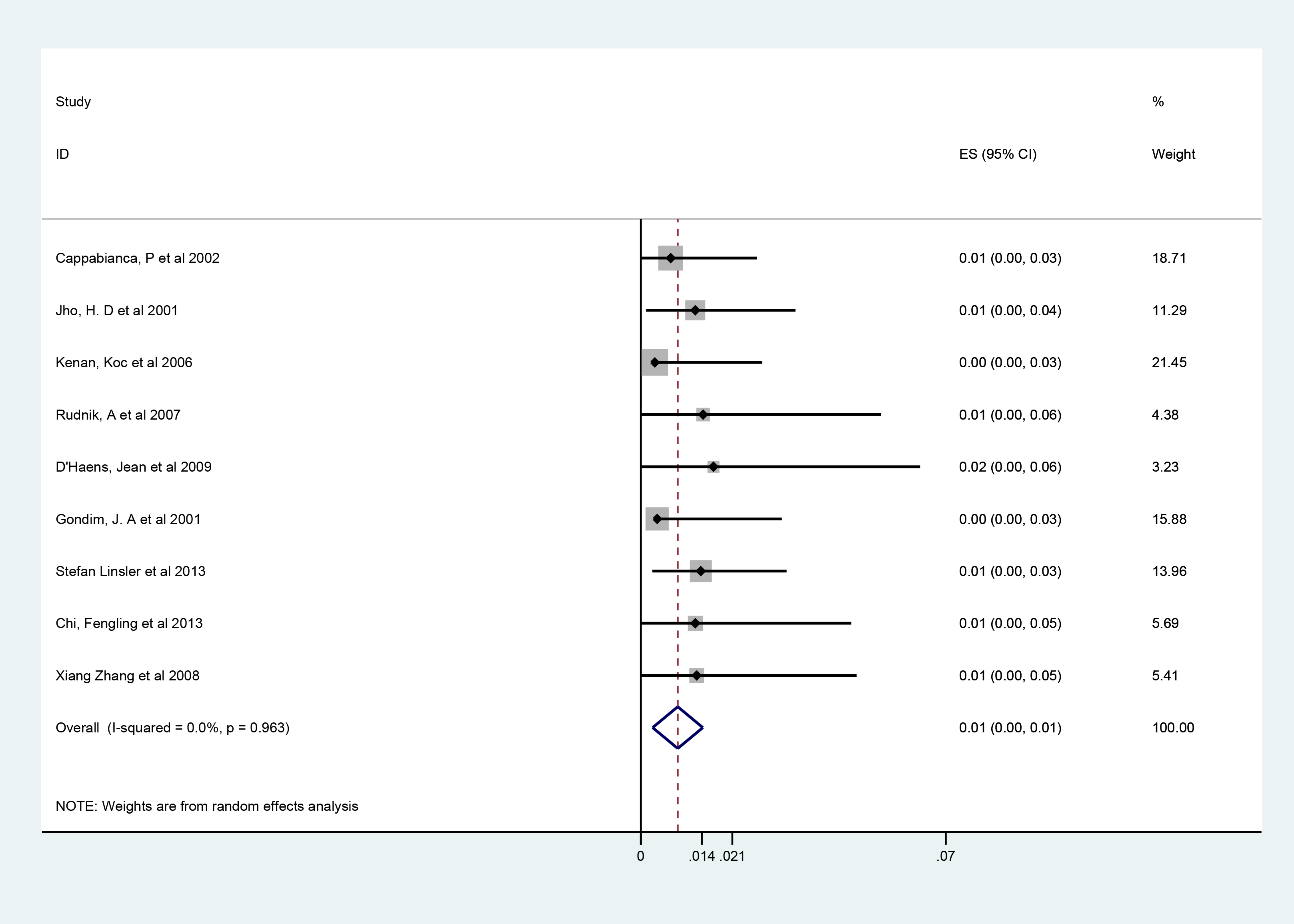

Supplement: S24 Fig — (TIF) [file pone.0153397.s025.tif]

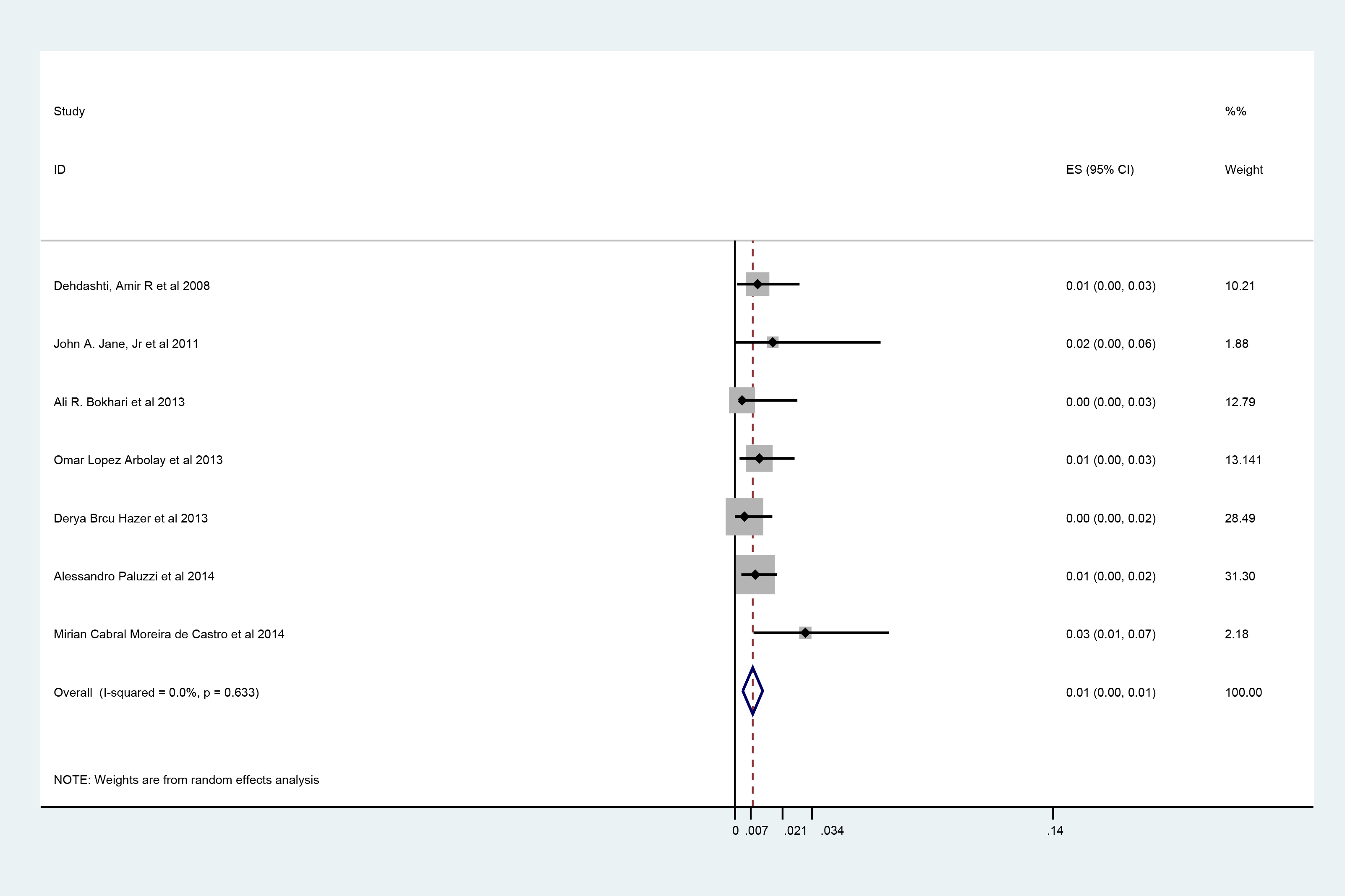

Supplement: S25 Fig — (TIF) [file pone.0153397.s026.tif]

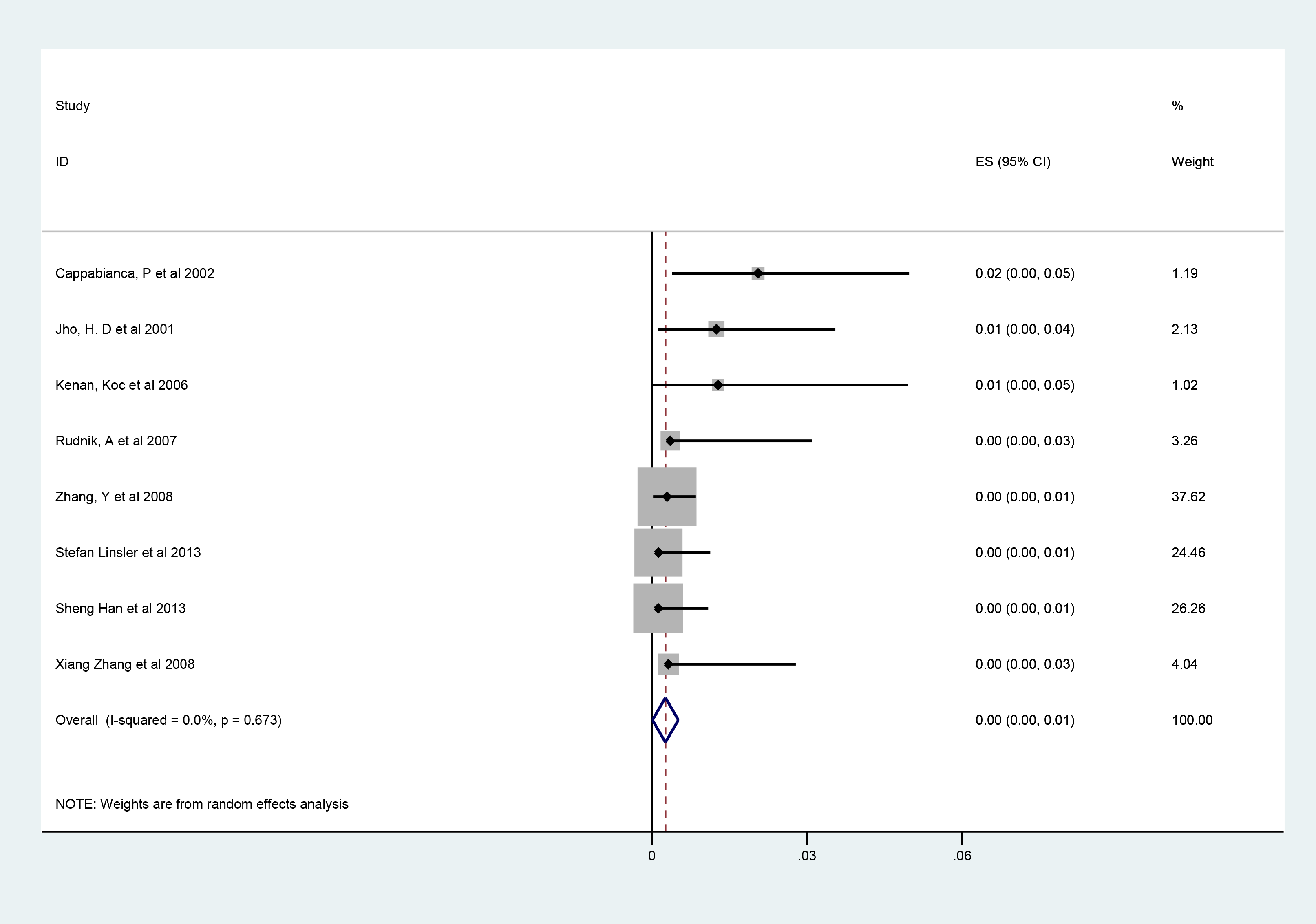

Supplement: S26 Fig — (TIF) [file pone.0153397.s027.tif]

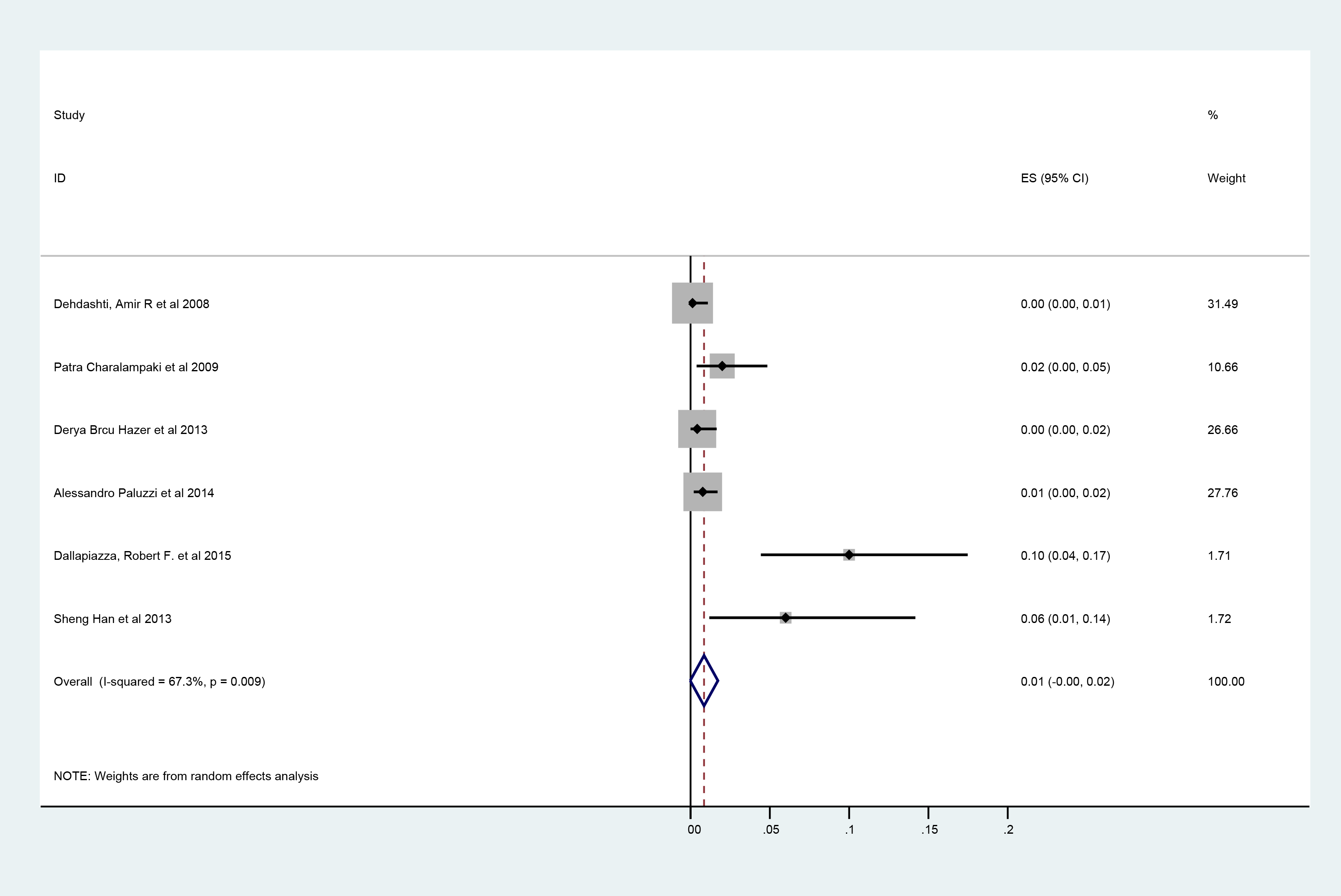

Supplement: S27 Fig — (TIF) [file pone.0153397.s028.tif]

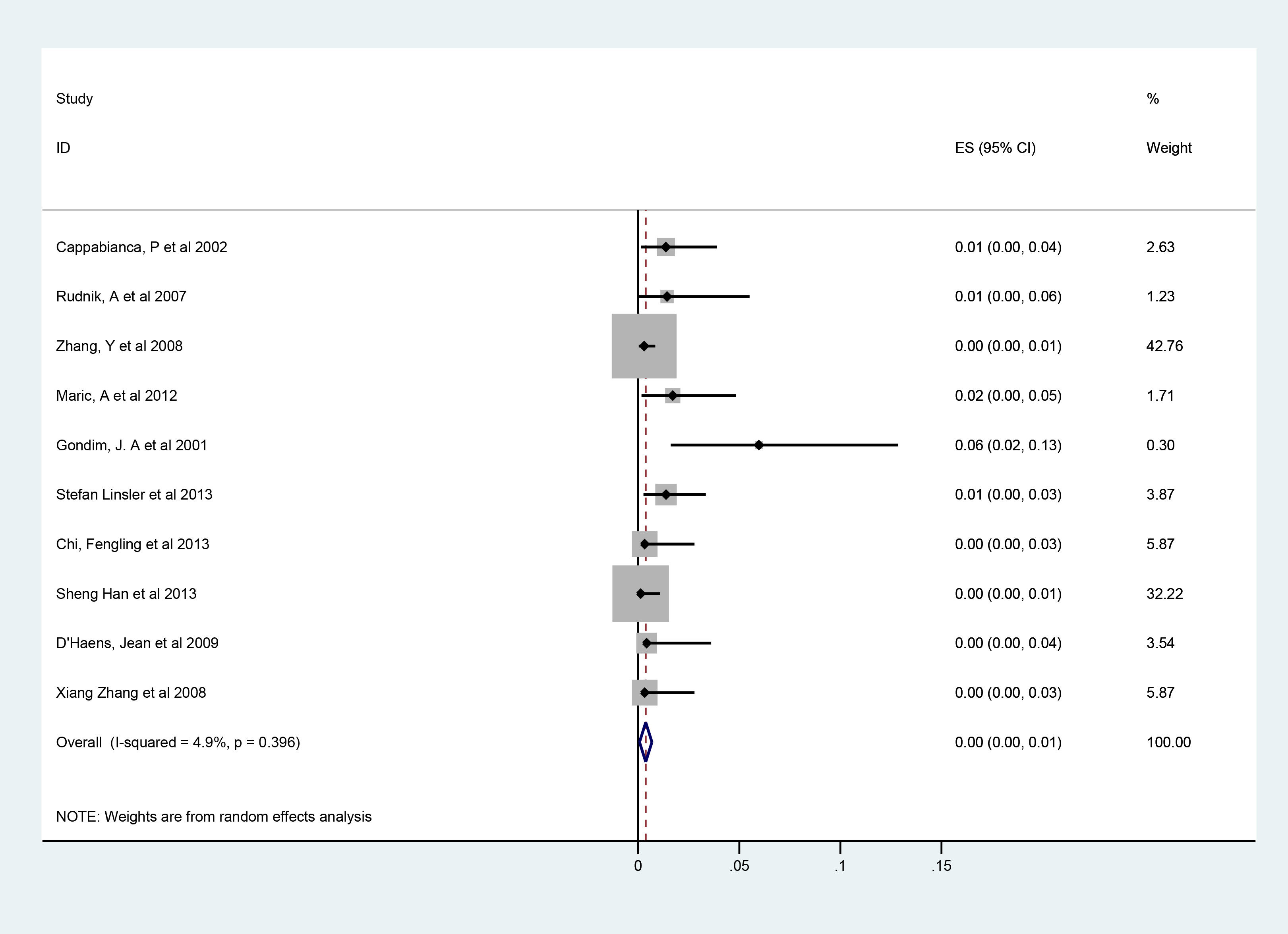

Supplement: S28 Fig — (TIF) [file pone.0153397.s029.tif]

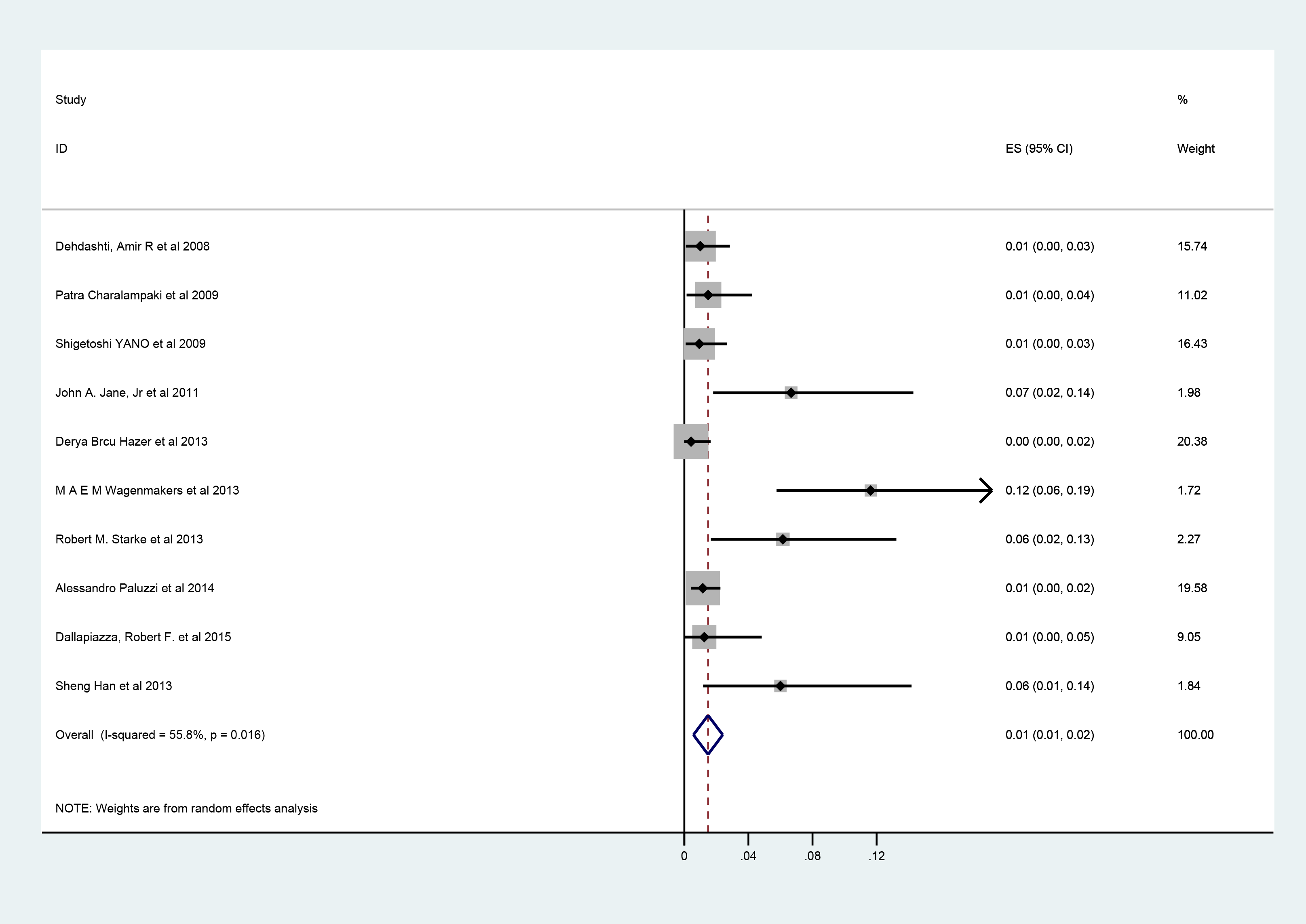

Supplement: S29 Fig — (TIF) [file pone.0153397.s030.tif]
