# Supplementary material for: Mononostril versus Binostril Endoscopic Transsphenoidal Approach for Pituitary Adenomas: A Systematic Review and Meta-Analysis
Source: PLoS One. 2016 Apr 28;11(4):e0153397. doi: 10.1371/journal.pone.0153397 (PMC4849742; doi:10.1371/journal.pone.0153397)
Supplement: S1 File — (DOC) [file pone.0153397.s031.doc]

**( lactotrophic adenoma OR lactotrophic tumor OR** [prolactinoma](javascript:void(0);) **OR PRL secreting adenoma OR PRL producing adenoma OR PRL secreting tumor OR PRL producing tumor OR thyrotrophic adenoma OR thyrotrophic tumor OR TSH secreting adenoma OR TSH producing adenoma OR TSH secreting tumor OR TSH producing tumor OR pituitary adenoma OR pituitary tumor OR pituitary neoplasm OR hypophysoma OR hypophyseal tumor OR hypophyseal adenoma OR acromegaly OR gigantism OR somatotrophic adenoma OR somatotrophic tumor OR GH secreting adenoma OR GH producing adenoma OR GH secreting tumor OR GH producing tumor OR Gonadotrophic adenoma OR gonadotrophic tumor OR LH secreting adenoma OR LH producing adenoma OR LH secreting tumor OR LH producing tumor OR FSH secreting adenoma OR FSH producing adenoma OR FSH secreting tumor OR FSH producing tumor OR non functioning adenoma OR non secreting adenoma OR non functioning tumor OR non secreting tumor OR null cell adenoma OR null cell tumor OR plurihormonal adenoma OR multihormonal adenoma OR plurihormonal tumor OR multihormonal tumor OR pituitary carcinoma ) AND ( Endoscopic OR Endoscope OR Endoscopes OR Endoscopy OR Endoscopies ) AND ( Trassphenoidal OR Transnasal-transsphenoidal OR Transoral-nosal-sphenoidal OR Transnasal-sphenoidal )**
