# Supplementary material for: Mononostril versus Binostril Endoscopic Transsphenoidal Approach for Pituitary Adenomas: A Systematic Review and Meta-Analysis
Source: PLoS One. 2016 Apr 28;11(4):e0153397. doi: 10.1371/journal.pone.0153397 (PMC4849742; doi:10.1371/journal.pone.0153397)
Supplement: S1 Table — (DOC) [file pone.0153397.s032.doc]

S1 table. The characteristic of the included studies.

1. Yano S, Kawano T, Kudo M, Makino K, Nakamura H, Kai Y, et al. Endoscopic Endonasal Transsphenoidal Approach Through the Bilateral Nostrils for Pituitary Adenomas. Neurologia Medico-Chirurgica. 2009; 49: 1-6.
2. El-Fiki ME, Aly A, Elwany S. Binasal Endoscopic Approach to the Sellar Region: Experience and Outcome Analysis of 80 Cases. Journal Of Neurological Surgery Part B-Skull Base. 2012; 73: 287-291.
3. Bokhari AR, Davies MA, Diamond T. Endoscopic transsphenoidal pituitary surgery: a single surgeon experience and the learning curve. British Journal Of Neurosurgery. 2013; 27: 44-49.
4. Mamelak AN, Carmichael J, Bonert VH, Cooper O, Melmed S. Single-surgeon fully endoscopic endonasal transsphenoidal surgery: outcomes in three-hundred consecutive cases. Pituitary. 2013; 16: 393-401.
5. Wagenmakers MAEM, Boogaarts HD, Roerink SHPP, Timmers HJLM, Stikkelbroeck NMML, Smit JWA, et al. Endoscopic transsphenoidal pituitary surgery: a good and safe primary treatment option for Cushing's disease, even in case of macroadenomas or invasive adenomas. European Journal Of Endocrinology. 2013; 169: 329-337.
6. de Castro MCM, Michel LMP, Denarol MMD, Gontijo PAM, de Sousa AA. Endoscopic transnasal approach for removing pituitary tumors. Arquivos De Neuro-Psiquiatria. 2014; 72: 378-382.
7. Paluzzi A, Fernandez-Miranda JC, Stefko ST, Challinor S, Snyderman CH, Gardner PA. Endoscopic endonasal approach for pituitary adenomas: a series of 555 patients. Pituitary. 2014; 17: 307-319.
8. Dubey SP, Munjal VR. Endoscopic endonasal transsphenoidal hypophysectomy: two hand versus four hand technique: our experience. Indian J Otolaryngol Head Neck Surg. 2014; 66: 287-290.
