# Supplementary material for: Mononostril versus Binostril Endoscopic Transsphenoidal Approach for Pituitary Adenomas: A Systematic Review and Meta-Analysis
Source: PLoS One. 2016 Apr 28;11(4):e0153397. doi: 10.1371/journal.pone.0153397 (PMC4849742; doi:10.1371/journal.pone.0153397)
Supplement: S2 Table — (DOC) [file pone.0153397.s033.doc]

S2 Table. Patient characteristics.

| Patient Characteristics | mon | bi | *p value (mon vs bi) |
| --- | --- | --- | --- |
| Total patients | 2285 | 2580 | / |
| Mean age | 45.5 | 48.7 | 0.164 |
| Mean follow-up | 30.9 | 35.1 | 0.575 |
| Male (%) | 39.4 | 44 | 0.199 |
| Macroadenomas (%) | 74.9 | 83.1 | 0.12 |
| mon, mononostril ; bi, binostril. *Statistical analysis performed using T- test. | | | |
