# Supplementary material for: Mononostril versus Binostril Endoscopic Transsphenoidal Approach for Pituitary Adenomas: A Systematic Review and Meta-Analysis
Source: PLoS One. 2016 Apr 28;11(4):e0153397. doi: 10.1371/journal.pone.0153397 (PMC4849742; doi:10.1371/journal.pone.0153397)
Supplement: S3 Table — (DOC) [file pone.0153397.s034.doc]

S3 Table. The length of hospital stay.

| The length of hospital stay  between mononostril and binostril appraoch | | | |
| --- | --- | --- | --- |
|  | mon | bi | P value* |
| Mean time , day | 4.4 | 3.2 |  |
| Range | 1.6-6.5 | 2.3-4.7 |
| Number of studies | 4(584)↑ | 4(493)↑↑ | 0.00 |
| *Statistical analysis performed using Mann-Whitney U test. ↑References 11,16,19,37;↑↑References 22,26,34,37. | | | |
